# Acceptance and Commitment Training for Parents of Children With Autism Spectrum Disorder: A Randomized Clinical Trial

**DOI:** 10.1001/jamanetworkopen.2025.52693

**Published:** 2026-01-08

**Authors:** Si Ni Li, Wai Tong Chien

**Affiliations:** 1 Xiangya School of Nursing, Central South University, Changsha, China; 2 The Nethersole School of Nursing, Faculty of Medicine, The Chinese University of Hong Kong, Hong Kong

## Abstract

**Importance:**

Parents of children with autism spectrum disorder (ASD) experience significant stress and caregiving burden and urgently require targeted psychological support and parenting guidance. Integrating acceptance and commitment therapy (ACT) into the World Health Organization’s caregiver skills training has demonstrated good feasibility, acceptability, and potential benefits in addressing these parents’ unmet psychological and informational needs; however, its effectiveness remains unexplored.

**Objective:**

To evaluate the effectiveness of an evidence-based ACT-based parenting program on parental stress and that of other health outcomes on parents and their children with ASD immediately and 6 months after intervention.

**Design, Setting, and Participants:**

This randomized clinical trial using the intention-to-treat principle was conducted across 7 government-designated rehabilitation institutions in Shenzhen, China, from February 18, 2024, to January 20, 2025. Participants were adult parents serving as the primary caregivers of their children with ASD (aged 3-9 years).

**Interventions:**

Participants were randomly allocated in a 1:1 ratio to either an intervention group or a control group using block randomization. Participants in the intervention group received usual care plus an 8-week, group-format ACT-based parenting program, while the control group received usual care only.

**Main Outcomes and Measures:**

Parental stress was the primary outcome, assessed by the Chinese version of the Parenting Stress Index–Short Form. Secondary outcomes included parental depressive symptoms, anxiety, psychological flexibility, and parenting competence and children’s emotional and behavioral problems.

**Results:**

Among 154 parents (mean [SD] age, 36.55 [4.92] years; 135 [87.66%] mothers) caring for children with ASD (mean [SD] age, 5.69 [1.75] years; 118 [76.62%] boys), 77 participants were randomized to the ACT-based intervention group and 77 were randomized to the control group. Those in the intervention group reported significantly greater improvements in parental stress (group × time effect, β = −2.04 [95% CI, −3.51 to −0.57]; *P* = .007), psychological flexibility (β = 1.12 [95% CI, 0.29 to 1.95]; *P* = .008), and parenting competence (β = 2.45 [95% CI, 0.53 to 4.36]; *P* = .01) and children’s emotional and behavioral problems (β = −1.16 [95% CI, −2.26 to −0.05]; *P* = .04) during the 6-month follow-up. Significant effects on parental depressive symptoms (β = −1.61 [95% CI, −3.12 to −0.10]; *P* = .04) and anxiety (β = −1.62 [95% CI, −2.81 to −0.44]; *P* = .007) were observed in the ACT-based intervention group immediately post intervention.

**Conclusion and Relevance:**

In this randomized clinical trial of an ACT-based parenting program, the intervention was effective in helping parents manage their stress in caregiving and the emotions and behaviors of their children with ASD, underscoring the need for future studies among more diverse populations globally.

**Trial Registration:**

ChiCTR Identifier: 2400080472

## Key Points

**Question:**

What is the effect of an acceptance and commitment therapy (ACT)–based parenting program on parental stress among parents of children with autism spectrum disorder (ASD)?

**Findings:**

In this randomized clinical trial including 154 participants, the ACT-based parenting program plus usual care led to greater 6-month reductions in parental stress and in the emotional and behavioral problems of children with ASD, as well as improvements in psychological flexibility, parenting competence, and reduced parental depressive symptoms and anxiety. Reductions in parental depressive symptoms and anxiety were evident only in the immediate postintervention period when compared with usual care alone.

**Meaning:**

These findings show that ACT-based parenting program was effective in reducing parental stress, suggesting that further testing in more diverse groups of parents is warranted.

## Teaser

This randomized clinical trial evaluates the effectiveness of the acceptance and commitment therapy–based parenting program on parental depressive symptoms, anxiety symptoms, psychological flexibility, and parenting competence as well as the emotional and behavioral problems of their children with autism spectrum disorder.

## Introduction

Autism spectrum disorder (ASD) is a heterogeneous neurodevelopmental condition characterized by persistent deficits in social communication and interaction, along with repetitive, restrictive, and inflexible patterns of behavior or interests.^1^ It is a prevalent neurodevelopmental condition in children, affecting approximately 12.07 million globally.^2^ The condition impacts children’s independent living, educational attainment, employment, skill acquisition, and social integration,^3^ thereby reducing their quality of life^4^ and significantly affecting the well-being of family caregivers, particularly parents.^5^

Parents of children with ASD often experience heavy psychological and caregiving burdens, with cross-sectional studies reporting parental stress prevalence rates ranging from 37% to 80% across various countries.^6,7,8^ Without adequate resources and social support, parental stress can escalate into severe psychological distress (ie, anxiety and depressive symptoms),^9,10^ further impairing parents’ ability to effectively support their children with ASD and attend to their own self-care needs.^11,12^

To support parents and their children with ASD, several parent-focused interventions have been developed to equip parents with knowledge and/or skills to manage their psychological experiences and respond flexibly to their children’s needs.^13^ However, these interventions showed limitations, including offering only emotional or informational support that fails to address parents’ diverse needs, lacking a theoretical framework to guide intervention development, and yielding inconsistent findings regarding their medium- to long-term sustainable effects.^14^ To address these limitations, we developed an acceptance and commitment therapy (ACT)–based parenting program, informed by evidence including our systematic review and meta-analysis,^15^ theoretical model of stress in families of children with developmental disabilities,^16^ *The Chinese Guideline of Treatment and Rehabilitation for Children With Autism*,^17^ ACT guidebooks,^18,19^ and the manual for World Health Organization caregiver skills training (WHO-CST).^20^ ACT uses 6 domains (ie, acceptance, cognitive defusion, contact with the present moment, self as context, values, and committed action) to facilitate psychopathology and behavior changes and thereby improve parents’ psychological flexibility during daily caregiving.^21^ The WHO-CST integrates naturalistic developmental behavioral interventions—widely regarded as the most promising approach for enhancing children’s reciprocal interactions and communication^22^—with education on disease management and self-care, equipping parents to understand their children’s needs, engage them in daily routines, and support their social, behavioral, and emotional development.^23,24^ By integrating these 2 potentially optimal intervention approaches identified in our systematic review and meta-analysis,^15^ this program fosters acceptance, mindfulness, and compassion to help parents manage negative emotions and experiences, clarify their parenting values and priorities, and develop skills to effectively address their children’s needs, conditions, and behaviors.^25,26^ Our pilot randomized clinical trial (RCT)^27^ demonstrated the program’s acceptability, feasibility, and preliminary effects in improving parental stress, depressive symptoms, anxiety symptoms, psychological flexibility, parenting competence, and emotional and behavioral problems of their children with ASD immediately after the intervention among 40 parents of children with ASD in China. The program’s feasibility and potential benefits highlight the need for a full-scale RCT with follow-up to assess its effectiveness and inform future clinical practice and research applications. Therefore, this study aimed to evaluate the effectiveness of the ACT-based parenting program on parental stress (primary outcome), depressive symptoms, anxiety symptoms, psychological flexibility, and parenting competence as well as the emotional and behavioral problems of their children with ASD, at baseline, immediately post intervention, and 6 months post intervention, compared with usual care only. Additional details on background context, intervention development and components, pilot trial and findings, and the present trial’s protocol and statistical analysis plan are provided in Supplement 1.

## Methods

### Study Design and Setting

This study was a parallel-group, 2-arm RCT with repeated measures conducted from February 18, 2024, to January 20, 2025, at 7 government-designated institutions in Shenzhen, Guangdong Province, China. These institutions specialize in providing rehabilitation services for children with developmental delays or disabilities. The Joint Chinese University of Hong Kong–New Territories East Cluster Clinical Research Ethics Committee approved the trial protocol. All participants provided written informed consent. The study adhered to the Consolidated Standards of Reporting Trials (CONSORT) reporting guideline.

### Participants

Eligible participants included 1 adult parent per family who was the primary caregiver of a child with ASD, aged 3 to 9 years, could read study materials, and had mobile or computer access for online sessions and surveys. When both parents were eligible, one was designated to avoid dyadic dependence. Exclusions were severe medical conditions precluding participation, unstable behaviors (eg, self-injury or suicidal behavior), caregiving for another acutely or chronically ill family member, planned or imminent hospital admission for the child, prior participation in an ACT-based parenting program, or current and/or recent participation in another parent support intervention.

The sample size (N = 154) was determined using G*Power, version 3.1 (Heinrich Heine University), based on a conservative a priori assumption of a medium effect size (Cohen *d* = 0.5) for the primary outcome (parental stress) and an attrition rate of 16.9%. These estimates were informed by data from 5 RCTs involving similar parents with 6-month follow-up periods, as identified in a previously published systematic review.^15^

### Randomization, Blinding, and Masking

Participants were randomized 1:1 to an ACT-based parenting program (n = 77) or to usual care (n = 77) using an online block randomization tool (block sizes of 4 or 6).^28^ The sequence was generated by a research assistant blinded to recruitment and data collection. Allocation concealment used sealed, opaque envelopes maintained by the same assistant, who opened them sequentially after consent. Blinding of interventionists and participants was infeasible given the intervention; participants were instructed not to disclose allocation. Because all outcomes were self-reported, outcome assessors (ie, participants) were not blinded.

### Interventions

Control participants received usual care from rehabilitation institutions, including at least twice-yearly public education on ASD rehabilitation and education on psychological health for parents and children, with crisis referral as needed. The intervention group received usual care plus 8 weekly 2-hour sessions of an ACT-based parenting program spanning 4 domains: emotion and stress management (ACT), parenting skills training, ASD-related education, and self-care. Delivery used a hybrid format: the first 4 sessions in person and the final 4 online, in groups of 6 to 8, to combine advantages of face-to-face interaction (direct observation, contextualized therapeutic work, and rapid rapport) with those of online delivery (accessibility, flexibility, and cost-effectiveness).^29,30,31^ Sessions used didactic instruction with slide presentations, ACT techniques (eg, metaphors, role play, experiential mindfulness exercises), and validated animated videos to support application of ACT strategies and parenting skills in daily caregiving. Parents received a guidebook summarizing key content, review points, and homework for each session and QR codes linking to online mindfulness practice videos; a detailed session outline appears in Supplement 1.

The intervention was delivered in accordance with the protocol and facilitated by a registered nurse specializing in psychiatric nursing with extensive experience supporting families of children with ASD in clinical settings (S.N.L.). To promote fidelity, the registered nurse completed ACT and WHO-CST training, obtained certifications, and conducted session-by-session case-scenario rehearsals before program launch. All sessions were audiorecorded and independently reviewed by an ACT supervisor—a nationally certified counselor in China—using the ACT Fidelity Checklist.^32^

### Data Collection Procedure and Measurements

Recruitment information was disseminated via institutional group text messages; interested parents completed QR code–linked online screening. Eligible individuals were contacted by a research assistant for an in-person visit to review objectives, procedures, risks and benefits, and rights and to address questions.

Baseline sociodemographic and clinical data were collected for parents and children. Outcomes were assessed at baseline (T0), immediately after the intervention (T1), and at 6 months (T2). Data were captured on WJX.cn, a widely used research platform in China with encryption and user authentication to support confidentiality and secure storage.

The primary outcome was parental stress as measured with the Parenting Stress Index–Short Form.^33^ Secondary outcomes included parental depressive symptoms (Patient Health Questionnaire–9),^34^ anxiety symptoms (Generalized Anxiety Disorder–7),^35^ psychological flexibility (Psy-Flex-C),^36^ parenting competence (Parenting Sense of Competence Scale),^37^ and the emotional and behavioral problems of their children with ASD (Strengths and Difficulties Questionnaire).^38^ All measures demonstrated good reliability (Cronbach α, 0.81-0.91) and were validated in Chinese populations. Detailed descriptions of sociodemographic and clinical variables and instrument information are provided in Supplement 1.

### Statistical Analysis

Data analysis was conducted using R software, version 4.4.1 for Macintosh (R Project for Statistical Computing), with a 2-sided significance level of *P* < .05. Normality of continuous variables was assessed using skewness and kurtosis statistics and Q-Q plots (eFigures 10-15 in Supplement 2). Baseline group differences used independent *t* tests for continuous variables and continuity-corrected χ^2^ or Fisher exact tests for categorical variables. Longitudinal outcomes were analyzed under the intention-to-treat principle^39^ using generalized estimating equations (GEE) to estimate population-averaged effects with robust inference for correlated measures. GEE models included fixed effects for group, time (categorical: T0, T1, and T2), and group × time interaction and were adjusted for prespecified covariates with baseline *P* < .10. Tukey-adjusted post hoc pairwise comparisons of model-derived marginal means estimated between-group differences at T1 and T2, controlling familywise error (eTable 2 in Supplement 2). Detailed specifications and all pairwise comparison results for parent and child outcomes are presented in eTable 2 in Supplement 2. The GEE quasi-likelihood approach yields unbiased treatment estimates under missing completely at random.^40^ Effect sizes (Cohen *d*) at T1 and T2 were interpreted as small (0.2 to <0.5), medium (0.5 to <0.8), and large (≥0.8).^41^

## Results

### Participant Enrollment and Sociodemographic and Clinical Characteristics

Among 275 parents screened for eligibility, 174 met the inclusion criteria. Of these, 154 parents provided informed consent and were randomly assigned to 1 of the 2 study groups after baseline assessment, with 77 participants randomized to the intervention group and 77 randomized to the control group (Figure). One participant withdrew at the 6-month follow-up assessment due to hospitalization, resulting in a 0.65% dropout rate. In the intervention group (n = 77), 46 participants (59.74%) completed all 8 sessions, and 71 (92.20%) completed 6 or more sessions.

**Figure.**
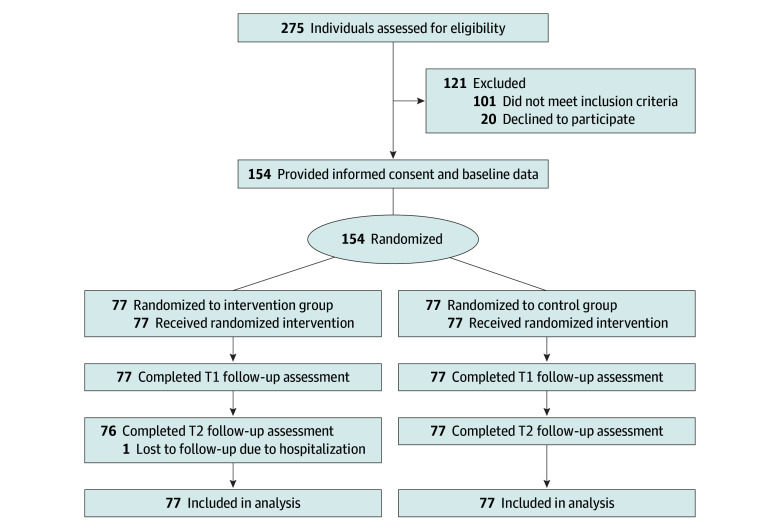
Study Flowchart One intervention-group participant missed the 6-month (T2) assessment due to hospitalization but was retained in the intention-to-treat analysis with available data. T1 indicates immediate postintervention assessment.

Participants (mean [SD] age, 36.55 [4.92] years) were all of Asian (Chinese) ethnicity, predominantly female (135 [87.66%] compared with 19 [12.34%] male), and married (143 [92.86%]). Their children (36 [23.46%] female and 118 [76.62%] male; mean [SD] age, 5.69 [1.75] years) had a mean (SD) duration of ASD diagnosis of 2.88 (1.52) years. Sociodemographic and baseline outcome variables showed comparability between groups (Table 1 and eTable 1 in Supplement 2). However, 4 covariates (parental employment, family income, child social interaction, and education type) required adjustment in the GEE analysis. The primary and secondary outcomes and the effect sizes are listed in Table 2; pairwise comparisons, in eTable 2 in Supplement 2; and longitudinal profiles, in eFigures 1 to 9 in Supplement 2.

**Table 1.** Comparisons of Sociodemographic and Clinical Characteristics of Participants Between Both Study Groups at Baseline Measurement^a^

Characteristic at baseline	All participants (N = 154)	Intervention group (n = 77)	Control group (n = 77)
**Parents**			
Age, mean (SD), y	36.55 (4.92)	36.88 (5.28)	36.20 (4.54)
Sex			
Female	135 (87.66)	68 (88.31)	67 (87.01)
Male	19 (12.34)	9 (11.69)	10 (12.99)
Marital status			
Married	143 (92.86)	71 (92.21)	72 (93.51)
Divorced or other	11 (7.14)	6 (7.79)	5 (6.49)
Educational level			
Secondary or below	20 (13.00)	8 (10.39)	12 (15.59)
Tertiary	123 (79.87)	62 (80.52)	61 (79.22)
Postgraduate or above	11 (7.14)	7 (9.09)	4 (5.19)
Employment status			
Full-time	65 (42.21)	39 (50.65)	26 (33.77)
Part-time	29 (18.83)	14 (18.18)	15 (19.48)
Unemployed or retired	60 (38.96)	24 (31.17)	36 (46.75)
Spouse’s or partner’s employment status			
Full-time	138 (89.61)	69 (89.61)	69 (89.61)
Part-time	5 (3.25)	3 (3.90)	2 (2.60)
Unemployed, retired, or other	11 (7.14)	5 (6.49)	6 (7.79)
Monthly household income, ¥^b^			
≤10 000	56 (36.36)	25 (32.47)	31 (40.26)
10 001-20 000	57 (37.01)	24 (31.17)	33 (42.86)
20 001-30 000	23 (14.94)	16 (20.78)	7 (9.09)
>30 000	18 (11.69)	12 (15.58)	6 (7.79)
Housing type			
Publicly owned	20 (12.99)	10 (12.99)	10 (12.99)
Privately owned	79 (51.30)	36 (46.75)	43 (55.84)
Rented or other	55 (35.71)	31 (40.26)	24 (31.17)
Household size, No. of people			
2-4	102 (66.23)	53 (68.83)	49 (63.64)
≥5	52 (33.77)	24 (31.17)	28 (36.36)
No. of chronic diseases			
0	125 (81.17)	66 (85.71)	59 (76.62)
1-2	27 (17.53)	10 (12.99)	17 (22.08)
≥3	2 (1.30)	1 (1.30)	1 (1.30)
Time spent on caring for their child with ASD per day, mean (SD) [range], h	9.80 (4.53) [4-18]	9.68 (4.53) [4-18]	9.92 (4.57) [4-17]
**Children with ASD**			
Age, mean (SD), y (range, 3-9 y)	5.69 (1.75)	5.47 (1.73)	5.91 (1.75)
Sex			
Male	135 (87.66)	68 (88.31)	67 (87.01)
Female	19 (12.34)	9 (11.69)	10 (12.99)
Duration of ASD, mean (SD) [range], y	2.88 (1.52) [0.5-7]	2.74 (1.50) [1-7]	3.01 (1.54) [0.5-6]
Level of social communication^c^			
1	60 (38.96)	36 (46.75)	24 (31.17)
2	62 (40.26)	30 (38.96)	32 (41.56)
3	32 (20.78)	11 (14.29)	21 (27.27)
Level of restricted, repetitive behaviors^c^			
1	122 (79.22)	62 (80.52)	60 (77.92)
2	31 (20.13)	15 (19.48)	16 (20.78)
3	1 (0.65)	0	1 (1.30)
No. of comorbidities			
0	58 (37.66)	33 (42.86)	25 (32.47)
1-2	84 (54.55)	38 (49.35)	46 (59.74)
≥3	12 (7.79)	6 (7.79)	6 (7.79)
Type of school currently attending			
Special education	53 (34.42)	15 (19.48)	38 (49.35)
Mainstream education	87 (56.49)	56 (72.73)	31 (40.26)
Other or not appliable	14 (9.09)	6 (7.79)	8 (10.39)
Time spent professional behavioral and skills training services per wk, mean (SD) [range], h	15.76 (11.07) [2-56]	15.44 (10.28) [2-56]	16.08 (11.86) [2-50]
Psychotropic drugs intake			
Yes	11 (7.14)	5 (6.49)	6 (7.79)
No	143 (92.86)	72 (93.51)	71 (92.21)
No. of hospital visits due to ASD in last year, mean (SD) [range]	1.57 (1.97) [0-15]	1.65 (1.64) [0-10]	1.49 (2.26) [0-15]
Cost of ASD treatment and health care services in last year, mean (SD) [range], ¥^b^	52 071.98 (48 960.19) [0-300 000]	57 493.64 (43 875.94) [0-160 000]	46 650.32.3 (53 301.79) [0-300 000]

Abbreviation: ASD, autism spectrum disorder.

^a^ Data are presented as the No. (%) of participants unless indicated otherwise.

^b^ 1 Chinese ¥ = 0.14 US $.

^c^ Level 1 indicates mild, required support; level 2, moderate, required much support; and level 3, severe, required very substantial support.

**Table 2.** Generalized Estimating Equations Models for the Comparison of Each Outcome Across Time Between the Intervention and Control Groups^a^

Outcome variable, measure	Participant group score, mean (SD)		Coefficient						Cohen *d*
			Group		Time		Group × time		
	Intervention	Control	β (95% CI)	*P* value	β (95% CI)	*P* value	β (95% CI)	*P* value	
**Primary outcome**									
Parental stress									
PSI-SF-15 Total score^b^									
T0	41.79 (10.62)	44.43 (10.81)	−3.37 (−6.77 to 0.03)	.05	−0.38 (−1.39 to −0.63)	.46	NA	NA	NA
T1	36.65 (8.77)	44.49 (9.73)	NA	NA	NA	NA	−5.21 (−7.57 to −2.84)	<.001	0.85
T2	36.95 (8.26)	43.66 (10.74)	NA	NA	NA	NA	−4.08 (−7.02 to −1.13)	.007	0.70
PSI-SF-15 Distress subscale^b^									
T0	13.22 (4.16)	14.16 (4.31)	−1.16 (−2.47 to 0.14)	.08	−0.34 (−0.75 to 0.08)	.11	NA	NA	NA
T1	11.60 (3.23)	13.84 (4.36)	NA	NA	NA	NA	−1.31 (−2.46 to −0.16)	.025^a^	0.58
T2	11.60 (3.40)	13.48 (4.30)	NA	NA	NA	NA	−0.95 (−2.20 to 0.31)	.139	NA
PSI-SF-15 Parent-Child Dysfunctional Interaction subscale^b^									
T0	11.92 (3.95)	12.84 (4.27)	−1.18 (−2.45 to 0.09)	.07	−0.033 (−0.41 to 0.34)	.87	NA	NA	NA
T1	10.05 (3.26)	12.70 (3.44)	NA	NA	NA	NA	−1.73 (−2.73 to −0.72)	<.001	0.79
T2	10.45 (3.07)	12.78 (3.52)	NA	NA	NA	NA	−1.40 (−2.55 to −0.26)	.02	0.71
PSI-SF-15 Difficult Child subscale^b^									
T0	16.65 (4.66)	17.43 (4.88)	−1.03 (−2.56 to 0.51)	.19	−0.01 (−0.51 to 0.48)	.96	NA	NA	NA
T1	15.00 (4.12)	17.95 (4.40)	NA	NA	NA	NA	−2.17 (−3.27 to −1.07)	<.001	0.69
T2	14.90 (3.95)	17.40 (4.80)	NA	NA	NA	NA	−1.73 (−3.22 to −0.24)	.02	0.57
**Secondary outcomes**									
Parental depressive symptoms, PHQ-9 score^c^									
T0	7.58 (4.83)	8.48 (5.92)	−1.15 (−2.80 to 0.50)	.17	−0.16 (−0.65 to 0.34)	.54	NA	NA	NA
T1	5.81 (4.60)	8.31 (6.26)	NA	NA	NA	NA	−1.61 (−3.12 to −0.10)	.04^a^	0.46
T2	5.81 (4.64)	8.17 (6.10)	NA	NA	NA	NA	−1.47 (−2.97 to 0.04)	.06	NA
Parental anxiety, GAD-7 score^d^									
T0	6.05 (4.37)	6.83 (5.08)	−1.46 (−3.05 to 0.12)	.07	0.02 (−0.48 to 0.52)	.94	NA	NA	NA
T1	4.00 (3.82)	6.40 (5.75)	NA	NA	NA	NA	−1.62 (−2.81 to −0.44)	.007	0.49
T2	4.94 (5.15)	6.87 (5.09)	NA	NA	NA	NA	−1.16 (−2.90 to 0.59)	.19	NA
Parental psychological flexibility, Psy-Flex-C score^e^									
T0	20.92 (4.59)	19.73 (4.69)	1.42 (−0.03 to 2.86)	.06	−0.020 (−0.65 to 0.61)	.94	NA	NA	NA
T1	22.45 (3.57)	19.62 (4.29)	NA	NA	NA	NA	1.64 (0.17 to 3.11)	.03^a^	0.72
T2	23.13 (3.53)	19.69 (4.06)	NA	NA	NA	NA	2.25 (0.59 to 3.91)	.003^a^	0.90
Parenting competence									
PSOC Total score^f^									
T0	62.92 (16.04)	61.25 (12.45)	2.84 (−1.71 to 7.38)	.22	0.20 (−0.84 to 1.23)	.71	NA	NA	NA
T1	67.90 (11.40)	61.14 (11.13)	NA	NA	NA	NA	5.08 (1.44 to 8.71)	.006	0.60
T2	68.21 (10.58)	61.64 (10.28)	NA	NA	NA	NA	4.90 (1.07 to 8.72)	.01	0.63
PSOC Efficacy subscale^g^									
T0	32.53 (7.80)	32.68 (7.17)	0.46 (−1.75 to 2.66)	.69	0.14 (−0.54 to 0.81)	.69	NA	NA	NA
T1	34.83 (5.21)	32.64 (5.50)	NA	NA	NA	NA	2.34 (0.16 to 4.51)	.04^a^	0.41
T2	35.32 (4.64)	32.95 (5.29)	NA	NA	NA	NA	2.52 (0.28 to 4.76)	.03^a^	0.48
PSOC Satisfaction subscale^h^									
T0	30.39 (11.04)	28.57 (9.39)	2.38 (−0.91 to 5.68)	.16	0.058 (−0.72 to 0.83)	.88	NA	NA	NA
T1	33.06 (7.82)	28.51 (7.99)	NA	NA	NA	NA	2.74 (0.10 to 5.39)	.04	0.58
T2	32.88 (7.57)	28.69 (7.62)	NA	NA	NA	NA	2.38 (−0.44 to 5.19)	.10	NA
Emotional and behavioral problems of children with ASD									
SDQ Total Difficulties score^i^									
T0	21.87 (6.17)	22.95 (6.06)	−1.67 (−3.62 to 0.28)	.09	−0.12 (−0.84 to 0.60)	.75	NA	NA	NA
T1	19.78 (5.83)	23.12 (4.52)	NA	NA	NA	NA	−2.26 (−3.87 to −0.65)	.006^a^	0.64
T2	19.32 (7.04)	22.71 (5.17)	NA	NA	NA	NA	−2.31 (−4.52 to −0.10)	.04^a^	0.55
SDQ Emotional Problems subscale score^i^									
T0	3.64 (2.43)	3.97 (2.62)	−0.70 (−1.46 to 0.067)	.07	−0.10 (−0.43 to 0.22)	.53	NA	NA	NA
T1	3.05 (2.33)	4.23 (2.13)	NA	NA	NA	NA	−0.84 (−1.67 to −0.01)	.05^a^	0.53
T2	2.69 (1.76)	3.77 (2.33)	NA	NA	NA	NA	−0.74 (−1.63 to 0.15)	.10	NA
SDQ Conduct Problems subscale score^i^									
T0	6.08 (2.51)	6.14 (2.26)	−0.22 (−0.96 to 0.51)	.55	0.01 (−0.18 to 0.19)	.95	NA	NA	NA
T1	5.08 (2.14)	5.97 (1.62)	NA	NA	NA	NA	−0.83 (−1.48 to −0.17)	.01^a^	0.67
T2	5.25 (2.59)	6.16 (1.47)	NA	NA	NA	NA	−0.84 (−1.60 to −0.09)	.03^a^	0.43
SDQ Hyperactivity-Inattention subscale score^i^									
T0	6.43 (2.10)	6.84 (1.92)	−0.54 (−1.21 to 0.09)	.11	0.04 (−0.25 to 0.32)	.79	NA	NA	NA
T1	5.84 (1.91)	6.88 (1.93)	NA	NA	NA	NA	−0.62 (−1.20 to −0.05)	.03^a^	0.54
T2	6.10 (2.08)	6.92 (1.76)	NA	NA	NA	NA	−0.40 (−1.21 to 0.40)	.33	NA -
SDQ Peer Problems subscale score^i^									
T0	5.73 (2.28)	5.99 (2.45)	−0.21 (−0.89 to 0.51)	.56	−0.06 (−0.35 to 0.23)	.70	NA	NA	NA
T1	5.81 (1.97)	6.03 (1.81)	NA	NA	NA	NA	0.04 (−0.73 to 0.81)	.92	NA
T2	5.29 (1.99)	5.87 (1.55)	NA	NA	NA	NA	−0.33 (−1.19 to 0.54)	.46	NA
SDQ Prosocial subscale score^j^									
T0	4.22 (2.77)	3.97 (2.71)	0.43 (−0.38 to 1.25)	.30	0.20 (−0.11 to 0.51)	.67	NA	NA	NA
T1	4.56 (1.96)	4.05 (2.09)	NA	NA	NA	NA	0.26 (−0.50 to 1.02)	.51	NA
T2	4.29 (2.33)	4.10 (2.46)	NA	NA	NA	NA	−0.07 (−1.01 to 0.88)	.89	NA
SDQ Externalizing score^i^									
T0	12.51 (4.54)	12.99 (4.10)	−0.76 (−2.11 to 0.59)	.27	0.05 (−0.38 to −0.47)	.84	NA	NA	NA
T1	10.92 (3.35)	12.86 (2.83)	NA	NA	NA	NA	−1.45 (−2.50 to −0.41)	.007^a^	0.63
T2	11.35 (4.28)	13.08 (2.37)	NA	NA	NA	NA	−1.25 (−2.66 to −0.17)	.08	NA
SDQ Internalizing score^i^									
T0	9.36 (3.51)	9.96 (4.21)	−0.91 (−2.11 to 0.29)	.14	−0.16 (−0.66 to 0.33)	.52	NA	NA	NA
T1	8.86 (3.58)	10.26 (2.98)	NA	NA	NA	NA	−0.81 (−2.00 to 0.39)	.19	NA
T2	7.97 (3.54)	9.64 (3.38)	NA	NA	NA	NA	−1.07 (−2.47 to 0.34)	.14	NA

Abbreviations: ASD, autism spectrum disorder; GAD-7, Generalized Anxiety Disorder-7; NA, not applicable; PHQ-9, Patient Health Questionnaire; PSI-SF-15, Parenting Stress Index–Short Form; PSOC, Parenting Sense of Competence scale; SDQ, Strengths and Difficulties Questionnaire; T0, baseline; T1, immediate postintervention assessment; T2, 6-month follow-up.

^a^ All analyses were adjusted for 4 covariates—parental employment, family income, child social interaction, and education type—due to baseline between-group differences.

^b^ Scored on a Likert scale ranging from 15 to 75, with higher scores indicating greater parental stress.

^c^ Scores range from 0 to 27, with higher scores indicating higher severity of depressive symptoms.

^d^ Scores range from 0 to 21, with higher scores indicating higher severity of anxiety disorders.

^e^ Scores range from 6 to 30, with higher scores indicating a greater psychological flexibility.

^f^ Scores range from 17 to 102, with higher scores indicating a higher sense of parenting competence.

^g^ Scores range from 8 to 48, with higher scores indicating a higher sense of self-efficacy.

^h^ Scores range from 9 to 54, with higher scores indicating a higher sense of satisfaction and comfort with the parenting role.

^i^ Scores range from 0 to 40, with higher scores indicating greater difficulty levels.

^j^ Scores range from 0 to 40, with higher scores indicating greater child strengths.

### Primary Outcomes

The intervention group experienced significantly greater reductions in parental stress (per their Parenting Stress Index–Short Form total scores) at both T1 (β = −5.21 [95% CI, −7.57 to −2.84]; *P* < .001; Cohen *d* = 0.85) and T2 (β = −4.08 [95% CI, −7.02 to −1.13]; *P* = .007; Cohen *d* = 0.70). These findings demonstrated medium-to-large effect sizes compared with the control group.

### Secondary Outcomes

At T1, depressive symptoms (β = −1.61 [95% CI, −3.12 to −0.10]; *P* = .04; Cohen *d* = 0.46) and anxiety (β = −1.62 [95% CI, −2.81 to −0.44]; *P* = .007; Cohen *d* = 0.49) were significantly reduced in the intervention group compared with controls, although effect sizes were small. However, no significant group differences were noted at T2.

Parental psychological flexibility showed significantly greater improvement in the intervention group at both T1 (β = 1.64 [95% CI, 0.17-3.11]; *P* = .03; Cohen *d* = 0.72) and T2 (β = 2.25 [95% CI, 0.59-3.91]; *P* = .003; Cohen *d* = 0.90), with medium-to-large effect sizes, when compared with the control group. Similarly, parenting competence improved significantly in the intervention group at both T1 (β = 5.08 [95% CI, 1.44-8.71]; *P* = .006; Cohen *d* = 0.60) and T2 (β = 4.90 [95% CI, 1.07-8.72]; *P* = .01; Cohen *d* = 0.63), displaying medium effect sizes.

Reductions in children’s emotional and behavioral problems (Strengths and Difficulties Questionnaire Total Difficulties score) were significantly greater in the intervention group at both T1 (β = −2.26 [95% CI, −3.87 to −0.65]; *P* = .006; Cohen *d* = 0.64) and T2 (β = −2.31 [95% CI, −4.52 to −0.10]; *P* = .04; Cohen *d* = 0.55). Effect sizes were medium compared with the control group.

## Discussion

In this RCT, an ACT-based parenting program was effective in improving parental stress, parents’ psychological flexibility, parenting competence, and the emotional and behavioral problems of their children with ASD at the 6-month follow-up, as well as reducing parental depressive symptoms and anxiety only immediately after the intervention. To our knowledge, this is the first study to develop a hybrid-modality, group-based program integrating ACT with parenting skills training to provide multifaceted support for parents of children with ASD. These findings contribute to the understanding of autism management and caregiver support and facilitate the evidence-based development of similar psychotherapy-based interventions in future health care practice and research.

The ACT-based parenting program significantly reduced parental stress for parents of children with ASD at T1 and T2, demonstrating its benefits to address multifaceted parental stress through emotional and informational support. By integrating ACT with WHO-CST, parents not only learn techniques to manage parenting challenges but also acquire parenting skills (eg, communication and interaction) to better understand their children’s needs, potentially further reducing stress and improving mental well-being. These findings align with our published systematic review and meta-analysis,^15^ which showed that parent-focused interventions, including ACT, significantly reduced parental stress, with large effects observed immediately after the intervention compared with control groups. Similarly, the RCT performed by Çiçek Gümüş and Öncel^42^ found that a 6-week, nurse-led ACT intervention significantly reduced parental stress immediately and at 3 months after the intervention among 60 Turkish parents of children with ASD and co-occurring mental disorders. These results underscore the potential for integrating ACT-based parenting programs into routine ASD management and family support and highlight the critical role of health care professionals, particularly nurses, in providing timely mental health care and support for parents during hospital treatment and community rehabilitation.

The reductions in parental depressive and anxiety symptoms at T1 were promising, likely benefiting from ACT’s ability to help parents detach from negative cognitive patterns contributing to these symptoms.^43^ However, the lack of sustained improvement at T2 aligns with previous findings, such as an RCT in 60 parents of children with ASD in Canada.^44^ That study reported that a 2-day ACT workshop could produce significant reductions in depressive symptoms immediately after the intervention, but such a significant effect could not be sustained to 10 weeks after the intervention.^44^ The result of nonsustainable improvement in our study might be due to the program’s lack of ongoing support and reinforcement (eg, booster sessions), highlighting the necessity of providing continuous support to help parents effectively navigate their long-term psychological challenges.^45,46^

Parental psychological flexibility improved significantly at both T1 and T2 in the intervention group, consistent with findings from the study by Çiçek Gümüş and Öncel,^42^ which demonstrated significant postintervention and 3-month improvements in psychological flexibility among parents of children with ASD and co-occurring mental disorders in Türkiye. ACT techniques in terms of its 6 domains (ie, acceptance, cognitive defusion, contact with the present moment, self-as-context, values, and committed action) improved parents’ psychological flexibility by helping them change their relationship with stressors (eg, encouraging acceptance of difficult emotions), break free from maladaptive cognitive and behavioral patterns (eg, cognitive fusion and experiential avoidance), clarify their parenting values and associated goals, and take value-based actions to improve self-care and caregiving, ultimately better supporting their children.^25^ However, Maughan et al^44^ reported that a 2-day intensive ACT workshop for 54 parents of children with ASD did not result in sustained improvements in psychological flexibility at 10 weeks after intervention.^44^ This discrepancy raises questions about the optimal duration and intensity of ACT interventions needed for lasting benefits. While short-term workshops might provide immediate significant effects, sustained improvement would require interventions with an adequate dose (eg, ≥4 sessions delivered weekly or biweekly) and comprehensive content (eg, ACT techniques and parenting skills training).^47^

The improvements in parenting competence and in the emotional and behavioral problems of children with ASD at both T1 and T2 could benefit from the component of WHO-CST that educates parents about ASD and provides parenting strategies. This enabled parents to respond more sensitively to their children’s needs, foster positive parent-child interactions, and effectively support behavior management and emotional regulation of their children with ASD. This finding aligns with that of an RCT involving 168 parents of children with asthma (aged 3-12 years)^48^ that integrated ACT with parenting training, resulting in significant improvements in parenting competence and children’s conditions immediately after the intervention and at 6-month follow-up. In contrast, an RCT using psychological therapy alone^49^ failed to enhance parenting competence among 82 parents of children with ASD immediately after the intervention and at 1-month follow-up. These results highlight the importance of combining tailored psychological support (ie, ACT) with parental education to address the emotional and practical needs of parents caring for children with ASD while also enhancing their own well-being.

### Limitations

This study has some limitations. First, the 6-month follow-up limits inferences about durability; longer follow-up is needed to assess sustained effects. Second, absence of postintervention booster sessions may have constrained consolidation and maintenance. Third, the sample was relatively homogeneous (predominantly married mothers, with narrow parent and child age ranges) and recruited by convenience from a highly developed metropolitan setting (Shenzhen), restricting generalizability to fathers, broader age groups, and less developed or rural contexts. Randomization was not stratified by ASD symptom severity, leaving potential residual imbalance; future trials should use more diverse sampling and stratified or covariate-adaptive randomization. Fourth, lack of participant blinding may have introduced performance, social desirability, and other subjective biases, threatening internal validity. Fifth, reliance on self-report may not capture objective change, limiting robustness; nonspecific influences (eg, expectancy, group cohesion) cannot be excluded. Future studies should include active controls matched on contact time, group interaction, facilitator characteristics, and session duration and incorporate standardized measures of nonspecific factors and objective outcomes. Last, the intervention combined ACT with core elements of WHO-CST; observed benefits reflect the package rather than ACT alone. Dismantling or factorial trials are needed to identify active components.

## Conclusions

In this RCT, the ACT-based parenting program was effective in improving parental stress, psychological flexibility, parenting competence, and the emotional and behavioral problems of children with ASD in a short- to medium-term follow-up period, as well as reducing parental depressive symptoms and anxiety immediately after the intervention. Future studies are recommended to include longer-term follow-ups to assess sustained effects, incorporate booster sessions to enhance ongoing support, include diverse participants and their children with ASD to improve generalizability, implement an active control group to enable blinding, use objective measures alongside self-reports to strengthen the robustness of the findings, and systematically explore how ACT components can be integrated into the WHO-CST framework to enhance its effectiveness across diverse cultural and contextual settings.

## Notes

Supplement 1.
Trial Protocol

Supplement 2. **eTable 1.** Study Outcome Scores of Participants and Their Children With ASD at Baseline **eTable 2.** Pairwise Comparisons of Mean Differences of the Outcome Scores for Both Parents and Their Children With ASD Between Groups at T1 and T2 **eFigure 1.** Profile Plot of Parental Stress of the Study Groups Across the 3 Time Points **eFigure 2.** Profile Plots of the 3 Subscales of Parental Stress of the Study Groups Across the 3 Time Points **eFigure 3.** Profile Plot of Parental Depressive Symptoms of the Study Groups Across the 3 Time Points **eFigure 4.** Profile Plot of Parental Anxiety of the Study Groups Across the 3 Time Points **eFigure 5.** Profile Plot of Parental Psychological Flexibility of the Study Groups Across 3 Measurements **eFigure 6.** Profile Plot of Parenting Competence of the Study Groups Across the 3 Time Points **eFigure 7.** Profile Plots of the 2 Subscales of Parenting Competence of Study Groups Across 3 Measurements **eFigure 8.** Profile Plot of Total Difficulties of Autistic Children’s Emotional and Behavioral Problems of the Study Groups Across the 3 Time Points **eFigure 9.** Profile Plots of the 8 Subscales of Autistic Children’s Emotional and Behavioural Problems of the Study Groups Across the 3 Time Points **eFigure 10.** The Q-Q Plot for the Total Score of Parenting Stress **eFigure 11.** The Q-Q Plot for the Total Score of Parental Depressive Symptoms **eFigure 12.** The Q-Q Plot for the Total Score of Parental Anxiety **eFigure 13.** The Q-Q Plot for the Total Score of Parental Psychological Flexibility **eFigure 14.** The Q-Q Plot for the Total Score of Parenting Competence **eFigure 15.** The Q-Q Plot for the Total Difficulties Score of Children’s Emotional and Behavioral Problems

Supplement 3.
Data Sharing Statement
