# Supplementary material for: Acceptance and Commitment Training for Parents of Children With Autism Spectrum Disorder: A Randomized Clinical Trial
Source: JAMA Netw Open. 2026 Jan 8;9(1):e2552693. doi: 10.1001/jamanetworkopen.2025.52693 (PMC12784228; doi:10.1001/jamanetworkopen.2025.52693)
Supplement: Supplement 1. — Trial Protocol [file jamanetwopen-e2552693-s001.pdf]

Trial Protocol

A randomized controlled trial of an acceptance and commitment therapy-based parenting program for improving parenting stress and other health outcomes of parents and children with autism

1 Introduction

1.1 Definition, prevalence, and disease burden of children with autism

Autism is a heterogeneous neurodevelopmental condition with a specific combination of persistent deficits in the ability to initiate/sustain reciprocal social communication and interaction, and by a range of repetitive, restrictive, and inflexible patterns of interests, behaviors, or activities.[1] Autism affects approximately 1% of the population globally,[2] one in 44 children aged below eight years in the United States,[3] and 1.8 million (0.7%) of Chinese children aged 6 to 12.[4] The presence of these autism-related conditions significantly affects their capacities for independent living, educational attainment, skill acquisition, and social integration,[5,6] which leads to a propensity for long-term dependency and challenges for their parents and other family caregivers.[7]

1.2 Needs and challenges of parents of children with autism

Parenting children with autism can be more stressful and challenging than parenting children with typical development.[8] Due to the constraints in healthcare, educational, and social services, parents of children with autism are obligated to fulfil dual roles as caregivers (e.g., coping with their children's autism-related conditions and comorbidities) and as partners in complex and intensive interventions (e.g., parent-mediated interventions).[9] As a result, these

parents had ongoing parenting stress and emotional turmoil (a set of negative and chronic emotional states such as guilt, grief, irritability, and pessimistic about the future) in managing their children's conditions/behaviors.[10] These emotional problems reduce parents' ability to sustain daily roles and increase the potential for developing mental health problems. Cross-sectional studies for parents of children with autism reported that 34.2% of them had clinically significant depressive symptoms, 44% had anxiety symptoms, and 55% had psychological distress.[11, 12] These emotional and mental health problems have been found to be associated with inadequate self-care practice,[13] inflexible parenting practices,[14] and reduced parenting competency.[15] These factors ultimately have a detrimental impact on their autism children's health outcomes.[16] Furthermore, it is common for parents of children with autism to express doubts regarding their own ability to recognize and comprehend the needs of their children with autism, as well as manage children's conditions and challenging behaviors.[9] For parents of children with autism, lower levels of parenting competency are associated with increased parenting stress and decreased motivation to participate in children's interventions.[17]

Therefore, given the high prevalence of mental health problems and unmet needs (e.g., limited access to mental health services and ASD-related knowledge) among parents of children with autism, it is crucial to develop a supportive intervention that not only assists these parents in improving their mental health and well-being but also provides them with the necessary knowledge and abilities to effectively address the conditions and behaviors of their children with autism.

2 Literature review and research gaps

2.1 Literature review on parental interventions

Traditional behavioural parent training, such as parent-mediated interventions, has been suggested to enhance the parental ability to manage children's conditions and behaviours, and promote parent-child interactions effectively.[18] However, these interventions have faced criticism for their narrow focus on the outcomes of children with autism, often neglecting the psychological difficulties experienced by parents of children with autism.[19] Consequently, an increasing number of parent-focused interventions have been developed to address this limitation and fulfil the needs of parents of children with autism. Parent-focused interventions primarily target parents' health outcomes such as mental health and well-being, and equip parents with knowledge and/or skills that enable them to effectively manage their psychological experiences and flexibly respond to their children's needs and conditions.[20] Various approaches have been developed and implemented in parent-focused interventions for supporting the parents of children with autism, such as cognitive behavioural therapy (CBT, aiming to identify and modify irrational emotions, cognitions, and behaviours), mindfulness-based interventions (MBIs, which aim to promote an individual's capability to be present in the moment without judgment), Acceptance and commitment therapy (ACT), psychoeducation (aiming to empower individuals with knowledge and skills to better manage and cope with mental health or behavioural challenges), among others. However, the effectiveness of parent-focused intervention in improving mental health and well-being for parents of children with autism were inconsistent and inconclusive because different approaches and formats used,

increasing the heterogeneity of the study groups/interventions and being unable to estimate the pooled effects of individual intervention approaches.[21]

2.2 Summary of a systematic review on parent-focused interventions

Therefore, a systematic review and meta-analysis was conducted to evaluate the effectiveness of their parent-focused interventions on promoting parents' mental health (e.g., stress, depressive symptoms, anxiety, and parental distress) and well-being (e.g., subjective well-being and parent-child relationships), as well as ameliorating children's autism symptoms and emotional and behavioral problems (EBPs), when compared with active/inactive controls.[22]

The systematic review identified a total of 21 RCTs using two-group, pretest-posttest design. The meta-analysis results showed that parent-focused interventions could reduce parents' stress (Hedge's g=-1.26), depressive symptoms (g=-0.71), and distress (g=-0.44), and children's EBPs (g=-0.21), and improved parent–child relationships (g=0.51) post-intervention, when compared with active/inactive control groups, with moderate to high certainty of evidence.[22] Moreover, ACT- and mindfulness-based intervention and parenting skills training were identified as the potential optimal intervention approaches to support autism parents' mental health and well-being in this systematic review and meta-analysis.

ACT. As a "third wave" of cognitive therapy, ACT composed of six interrelated processes/domains (acceptance, cognitive defusion, contact with the present moment, self-as-context, values, and committed action) and combines mindfulness principles with behavioral therapies to facilitate positive changes in psychopathology and behaviors in parallel with improving one's psychological flexibility.[23] ACT seems especially applicable in addressing the

emotional and mental health problems experienced by parents in caring for their children with autism. The preliminary effectiveness of ACT-based interventions in providing support for parents of children with autism was found consistent among four included studies, including one randomized controlled trial (RCT)[24] and three pilot-RCTs.[25-27] All of them were conducted using a face-to-face group format at weekly intervals, involving parents only, having "some concerns" risk of bias and small sample sizes (N<60 per study). The findings showed potential benefits of ACT-based intervention on several aspects of parents' psychological problems (e.g., parenting stress, distress, and depressive symptoms; Hedges' g ranged from 0.41 to 5.81) and well-being (e.g., psychological flexibility, mindful awareness, cognitive fusion, and personal value; g ranged from 0.39 to 6.08) post-intervention, when compared with either no treatment, treatment-as-usual or parent training group.

Parenting skills training. A component analysis reported that integrating psychological intervention (e.g., mindfulness-based intervention) with behavioral parenting skills training showed more favorable effect size than the psychological intervention alone (g=-0.83) and parenting skills training alone (g=-0.99) on parenting stress for parents of children with autism [28]. Parenting skills training targets parents' needs in acquiring autism-related knowledge, strategies and skills to enhance parent-child interaction and communication, and effective management of their children's conditions and behaviors.[29] By integrating ACT with parenting skills training, parents' requirements for self-care and attending to their children's conditions and behaviors could be met. Additionally, this approach could mitigate the psychological

difficulties that parents face when caring for children with autism and enhance their awareness and sensitivity to identify their children's development changes and needs.

Optimal intervention format. Optimal characteristics of parent-focused interventions, including targeted participants (only involving parents instead of parent-child dyads), intervention duration (5-8 weeks with weekly intervals), and delivery mode (group-based), were identified from the systematic review to inform the design of an ACT-based parental training intervention for improving these parents' mental well-being and their autism children's behavioral health outcomes.

2.2 Research gaps of existing autism-based interventions for parents of children with autism

First, only four experimental studies of ACT-based interventions for parents of children with autism were conducted. All of them were not high-quality RCT design (e.g., without allocation concealment and intervention adherence and pre-specified study protocol) and inadequate sample sizes and short follow-up periods (mainly pretest-posttest design); and they had inconsistent health outcomes (especially children's outcomes).

Second, the identified studies using ACT as the sole intervention approach focusing on parents' self-care but not caring for the parents' learning/training in caregiving needs and support for their children's development. As both parents' psychosocial health and their children's caring/behavioral needs are equally important, combining these two potential effective intervention approaches (ACT + parenting skills training) to be tested in a high-

quality RCT on both parents' and their autism children's psychosocial health outcomes is recommended.

Third, the ACT-based interventions were all performed by psychotherapists, such as psychologists and behavior analysts. When considering limited resources (e.g., time, cost, and staff) in parent-focused interventions, training non-specialist providers such as nurses and social workers to administer an ACT-based parenting program may be more feasible, applicable and even cost-effective in community care settings. For example, a parenting program developed by world health organization which training parents, nurses, and social works as primary care provider showed significantly improve on parental stress, competence, and parent support of interaction for Italian parents of children with ASD post-intervention and 3-month follow-up time points, as compared to the treatment-as-usual control group.[30]

Fourth, the delivery mode of all four studies identified was conducted face-to-face, and the feasibility and effectiveness of blended (combine the face-to-face and online modalities) ACT for parents of children with autism is still unknown. According to a recent RCT , there were no significant differences found between online and in-person ACT interventions for parents of children with typical development in terms of their effect on parents' stress levels, psychological flexibility, emotional regulation skills, and satisfaction with life post-intervention.[31] Indeed, online participation has several advantages, especially for parents of children with autism who need to balance multiple parenting roles. It eliminates time and space constraints, allows for better treatment adherence, and saves costs for both the service provider and parents.[32, 33] Likewise, face-to-face intervention allows direct observation and

identification of clinically relevant behaviors in therapy that help to contextualize and provide the client with the appropriate exercise or metaphor according to the clinical conversation.[34] And based on a previous RCT, ACT-based parenting skills training with a blended modality (2-session ACT plus 9-session parenting skills training) significantly reduced psychological distress and improved psychological flexibility, confidence in managing behaviors, and family adjustment post-intervention for parents of children with acquired brain injury, compared with the care-as-usual group.[35] Therefore, considering the advantages of these two modalities and the confirmed feasibility and efficacy of ACT-based parenting training with a blended modality for parents of children with other developmental disabilities in adjusting parents/family outcomes, future studies are recommended to explore the effectiveness of blended ACT for parents of children with autism.

Finally, all the ACT-based interventions were conducted in Western countries and thus the findings might not be transferable/generalizable to the Chinese context, where there are very limited practice guidelines, policies, and resources for autism family care.

Therefore, it is proposed to develop and validate an ACT-based parenting training program, to test the effectiveness of the validated intervention with a rigorous RCT design for improving both parents' and their autism children's psychosocial health outcomes in mainland China.

3 Theoretical framework: Model of stress in families of children with developmental disabilities

The theoretical model of stress (Figure 1) in families of children with developmental disabilities,[36] which was adopted by a pilot RCT of ACT-based program,[27] has been used to

guide the intervention development. This model implied that the risk and protective factors, including personal resources, family system resources, informal social support, and formal supports in the model can moderate the relationships between stressors (related to children's characteristics and other life stressors) and parents' outcomes.

By applying the ACT-based parental training program, parents can address the unmet needs related to their personal well-being (e.g., emotional and stress management, self-care learning, and parenting skills training), family dynamics (e.g., parent-child relationship), and formal/informal support factors. After receive this program, parents would have better psychosocial adjustment and health outcomes such as lessening parenting stress and depressive and anxiety symptoms,[37] and enhancing parents' psychological flexibility,[38] parenting competency[39]), as well as capability to successfully manage their children's illness and related problematic behaviors (e.g., children's emotional and behavioral problems; EBPs).

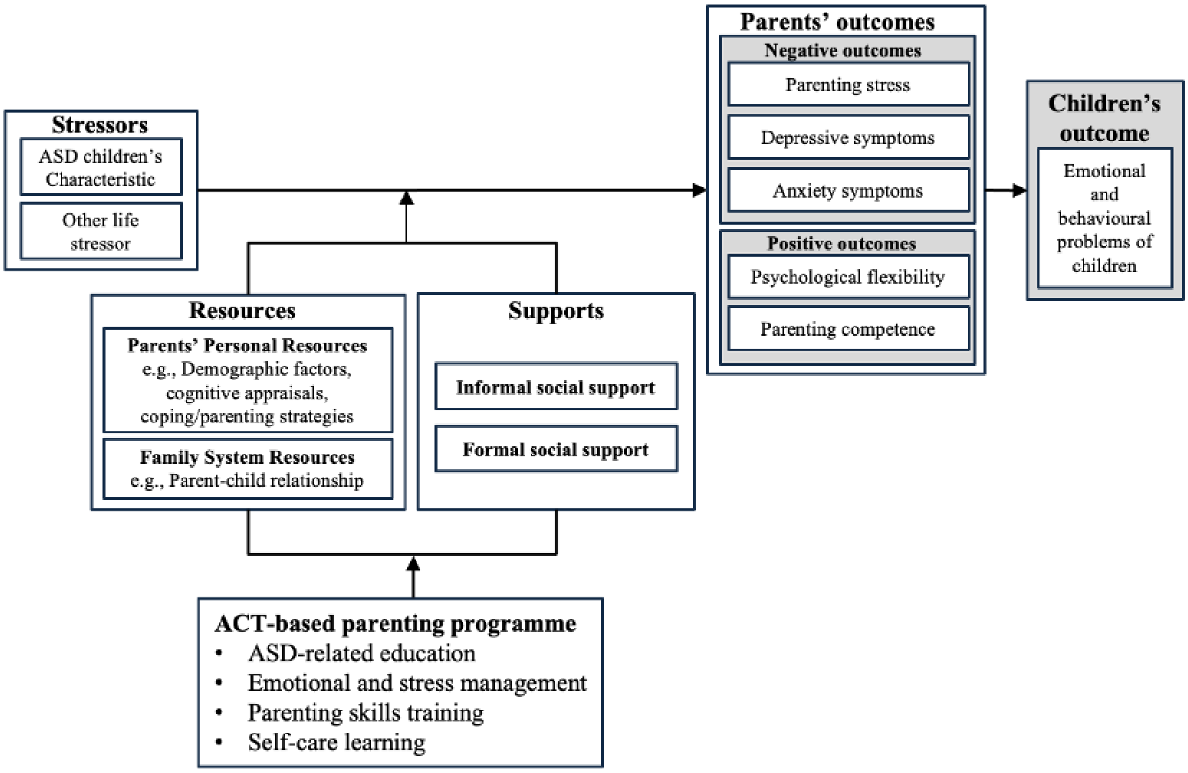

Figure 1 Adapted model of stress in families of children with developmental disabilities (Perry, 2004) to explain distress symptoms in parents of a child with autism. ASD = Autism spectrum disorder.

4 Operational definitions

Operational definitions of the terminologies or variables used in this study are presented as follows:

Children with autism. In this study, this term refers to children aged 3–9 years who received a clinical diagnosis of autism according to DSM-5 diagnostic criteria. We do not reference prior affiliated diagnoses that are no longer recognized as separate entities in the DSM-5 (e.g., autistic disorder, Asperger's disorder, pervasive developmental disorder–not otherwise specified, Rett syndrome, or childhood disintegrative disorder).[40] If historical documentation or clinical reports use these legacy terms, they were treated as referring to autism under DSM-5 nosology and were verified against DSM-5–based diagnostic confirmation for eligibility. The 3–9-year period spans preschool and early primary school, a critical window for development in social interaction and communication, adaptive behavior, cognition, and emotion—domains closely linked to core ASD characteristics.[41] Interventions during this period are particularly consequential for developmental outcomes. Moreover, children in these age stages often display more challenging behaviors than older age groups, which may contribute to elevated parenting stress.[42, 43]

Primary family caregiver. This term is operationally defined as the person who is living with their child with autism and takes the responsibility for coordinating caregiving activities,

consistently ensuring the provision of care and welfare for their children with autism, and typically dedicating the most time to fulfilling parental duties (at least four hours per day) [44].

5 Methods

5.2.1 Study aim and objectives

The aim of this study is to evaluate the effectiveness of ACT-based parenting program on parents' psychological health and their children's emotional and behavioural problems, when compared with usual-care-only.

The objectives of the main RCT are to assess the effects of ACT-based parenting program versus usual care on: a) parental outcomes: parenting stress (primary outcome), depressive symptoms, anxiety symptoms, psychological flexibility, and parenting competence; and b) children's outcome: emotional and behavior problems at immediately (T1) and six-month post-intervention (T2).

5.2.2 Research hypotheses for the main RCT

Compared with a usual-care-only group, parents in the ACT-based parenting program in addition to usual-care-only will indicate significantly greater improvement in parenting stress (primary outcome), depressive symptoms, anxiety symptoms, psychological flexibility, and parenting competence, and their children's emotional and behavior problems at immediately, and six-month post-intervention.

5.2.3 Study design

The main randomized controlled trial will employ a parallel-group, repeated-measures design and will adhere to the CONSORT (Consolidated Standards of Reporting Trials) guidelines [45].

5.2.4 Settings and Subjects

Parents will be recruited through seven rehabilitation institutions for children with developmental disabilities or delays in Guangdong Province, China. All seven institutions are located in urban districts of Shenzhen—a highly developed metropolitan area—and share comparable characteristics in organizational structure, funding support, service offerings, professional qualifications, and staff composition, representing services commonly provided by non-governmental institutions to children with ASD and their families in mainland China. Each institution is a privately operated, designated rehabilitation service provider authorized by the Shenzhen Disabled Persons' Federation and operates in accordance with government regulations.[46] Under Shenzhen municipal policies for rehabilitation assistance for children with disabilities,[47] intervention costs at these institutions can be subsidized by the government up to 50,000 RMB (approximately 7,021 USD) per child per year. The institutions included: Shenzhen Xinkangle Special Education and Rehabilitation Institution, Shenzhen Xingxinyuan Special Education and Rehabilitation Institution, Shenzhen Bougainvillea Special Needs Children's Rehabilitation Institution, Shenzhen Happy Nut Special Needs Children's Rehabilitation Institution, Shenzhen Love Ear Tree Special Needs Children's Rehabilitation Institution, Shenzhen Wishing Tree Special Needs Children's Rehabilitation Institution, and Shenzhen Stars Special Needs Children's Rehabilitation Institution. The first four sessions of

the intervention were administrated face-to-face at the children's rehabilitation institutions, and the last four sessions were conducted through an online videoconferencing platform (Tencent Meeting).

Participants (Parents) will be eligible if they are: (1) aged ≥ 18 years old; (2) parent of children aged between 3 to 9 years diagnosed with autism; (3) primary family caregivers responsible for daily care of their children with autism; (4) able to communicate and understand Mandarin; and (5) able to complete online sessions and questionnaires through mobile phone or computer.

Participants will be exclude if they had: (1) been diagnosed with clinically significant mental (e.g., anxiety disorders, depression, post-traumatic stress disorder, schizophrenia, developmental disorders) or medical conditions (e.g., spinal cord injury, spina bifida, cerebral palsy, multiple sclerosis, hearing/visual impairment) that prevented participation; (2) exhibited unstable or extreme intentions or behaviors (e.g., self-injury or suicide); (3) been caring for at least one other family member with an acute or chronic illness; (4) a planned or imminent hospitalization for their child with autism (including admission to an inpatient unit or long-term residential facility); and/or (5) been enrolled in another parent support intervention.

5.2.5 Sample size estimation

The effect size of parenting stress (primary outcome) was used to calculate the sample size. Our systematic review and meta-analysis found that the effect sizes of ACT-based interventions on reducing parenting stress in parents of children with autism were large, that is, Hedges' g=-1.25 (n=20) in Marion et al. (2021)[48] and d=-5.81 (n=60) in Çiçek & Öncel (2023)

[49] at immediately post-intervention when compared with parent training or treatment-as-usual control group, respectively.[22] The pooled effect size of parent-focused interventions in parents of children with autism also demonstrated a large effect size (Hedges' g=-1.26) on parenting stress immediately post-intervention, when compared with the active/inactive control groups. However, given very limited studies of ACT-based interventions and relatively small sample sizes used, a medium effect size of 0.5 (Cohen's d) on parenting stress was adopted to conservatively estimate the effects of this newly developed ACT-based parenting program for the parents caring for an autism child. Furthermore, attrition in RCTs can lead to biased treatment effect estimates, reduced statistical power, and limited generalizability [50]. To determine the appropriate sample size, the expected attrition rate must be considered. Five of the included studies[51-56] in the systematic review and meta-analysis had similar follow-up duration as this main RCT. The attrition rates ranged from as low as 0% [52-54] to as high as 59.65%.[51] Therefore, to mitigate the risk of decreased statistical power, an average attrition rate of 16.9% was considered for the main RCT. Finally, taking into account the possible attrition rate of 16.90%, a total of 154 participants (77 per study group) were required for a statistical power of 80% and statistical significance of 5%, two-tailed, using G*Power v. 3.1.9.7.[57]

5.2.6 Subject screening and recruitment

Teachers at each rehabilitation institution will assist in disseminating recruitment information to potential participants via text messages posted in school-affiliated WeChat groups. Parents who express interest will be invited to complete an online screening survey by

scanning a QR code provided on the recruitment information sheet. The screening will include questions (e.g., the child's age, respondent's daily caregiving hours) to assess eligibility against the study criteria. Following identification of potentially eligible parents, research assistants will contact them by telephone to introduce the study, assess interest, and schedule an in-person meeting. At the in-person meeting, research assistants will explain the study objectives, procedures, potential benefits and risks, and participants' rights (including the option to withdraw at any time). Written informed consent will be obtained after parents indicate full understanding and all questions have been addressed.

5.2.7 Randomization and concealment

Eligible participants will be randomly assigned in a 1:1 ratio to either an ACT–based parenting program plus usual care (intervention group) or usual care only (control group), using permuted block randomization with variable block sizes of four or six.[58] The allocation sequence will be generated by an online randomization program and administered by a research assistant (a registered nurse with a master's degree in nursing) who will not be involved in participant recruitment, intervention delivery, or outcome assessment. Group allocation will be concealed from outcome assessors, rehabilitation school teachers, parents, and children using sequentially numbered, opaque, sealed envelopes.

5.2.8 Intervention Group

In addition to usual care, parents in the intervention group will receive eight weekly sessions (2 hours per session) of an Acceptance and Commitment Therapy (ACT)–based parenting program delivered by the research team. The program will be administered in a

blended group format (face to face and online), with approximately 6–8 participants per group. The curriculum will cover four domains: (1) emotional and stress management (ACT); (2) autism-specific parenting skills; (3) autism-related education; and (4) self-care.

To establish rapport between the facilitator and participants, Session 1 will be delivered face to face. Sessions 2–4 will also be delivered face to face because they include experiential exercises (e.g., role-playing, mindfulness practices) that support learning of complex techniques and concepts. Sessions 5–8 will be delivered via an online videoconferencing platform (Tencent Meeting). During the final four online sessions, parenting practices will be taught step by step using instructional parenting videos, and core ACT principles (e.g., self-as-context) will be introduced using animated metaphor videos. All videos will be drawn from trustworthy, validated sources recommended by the World Health Organization's Caregiver Skills Training for Families of Children with Developmental Delays and Disabilities (WHO-CST)[30] and/or ACT manuals.[59, 60] Parents will receive a workbook outlining core ACT principles, key parenting skills, and weekly homework. Participants will be able to access online mindfulness practice videos after class by scanning QR codes provided in the workbook. A session-by-session outline of the ACT program will be provided in Table 1.

5.2.9 Control Group

Based on the government policy document titled "Management Measures for Designated Rehabilitation Service Institutions for Disabled Children and Youth in Shenzhen",[46] parents will receive usual care services from their children's rehabilitation institutions delivered by institutional staff (rehabilitation therapists, special educators, social workers with standard

local credentials) as part of routine, year-round services. To minimize nonspecific influences, both groups will have access to institution-based staff with comparable professional credentials and opportunities for contact and information provision. Usual care services will comprise in-person group education (~1 to 2 hours/session) and outreach activity (~3 to 4 hours/activity) held at least twice annually, as well as ad hoc individual consultations (~15 to 30 minutes/visit) covering autism-related rehabilitation education, interpretation of government support policies, or education on parental/child psychological health, with crisis referral when indicated). Usual care services will not include structured ACT content or the manualized WHO-CST curriculum; any overlap will be limited to general psychoeducation without skills training or fidelity-monitored components.

5.2.10 Measures

The instruments that will be used for outcome measurement in the RCT are described below.

Demographic questionnaire. A demographic data form was created to gather baseline information about parents and their children with autism. This form included details such as age, sex, family socioeconomic status, education level, and employment status. Moreover, it recorded children's medical characteristics, including the diagnosis dates for autism, any other medical conditions, and types of medications used in relation to autism.

Primary outcomes

The ***Chinese version of the Parenting Stress Index – Short Form (PSI-SF)*** is a 15-item self-reported questionnaire measuring parental stress by rating on a 5-point Likert scale (1 –

“strongly disagree” to 5 – “strongly agree”).[61] A higher score indicates a greater parental stress. This instrument was adopted from the original PSI-SF[62] (36-item; and designed to measure three domains: parental distress (PD), parent-child dysfunctional interaction (PCDI), and difficult child (DC). The Cronbach’s α of PSI-SF-15 was 0.87/0.86 (mother/father) for the total scale, 0.71/0.72, 0.82/0.78, and 0.79/0.78 for PD, PCDI, and DC, respectively,[61] confirming its good internal consistency in Chinese parents. The three-factor model with five items each was confirmed by confirmatory factor analysis (CFA) with an acceptable model fit and good structure validity.[61] No cut-off point for the Chinese version was reported.

Secondary outcomes for parents

The ***Patient Health Questionnaire (PHQ-9)*** is a nine-item self-reported questionnaire measuring depressive symptoms by rating on a 4-point Likert scale (0 – “Not at all” to 3 – “Nearly every day”) [63]. The higher scores indicate higher severity of depressive symptoms. The cut-off scores for this instrument were categorised as follows: mild depression (5-9), moderate depression (10-14), moderately severe depression (15-19), and severe depression (≥ 20).[64] The Chinese version of the PHQ-9 was validated in community adults and reported a good internal consistency reliability (α= 0.86) and a good one-factor structure (Kaiser–Meyer–Olkin= 0.89; the chi-square value of the Bartlett’s Test of Sphericity = 3453.80, p<.001).[64]

The ***Generalised Anxiety Disorder-7 (GAD-7)*** is a seven-item self-reported questionnaire measuring anxiety symptoms by rating on a 4-point Likert scale (0 – “Not at all” to 3 – “Nearly every day”).[65] The higher scores indicate higher severity of anxiety disorders. The cutoff points of this instrument were categorised as follows: none/normal (0-4), mild anxiety (5-9), moderate

anxiety (10-14), and severe anxiety (15-21).[66] The Chinese version of the GAD-7 was validated in outpatients from traditional Chinese internal departments and indicated an excellent internal consistency reliability (α= 0.91) and an adequate one-factor model fit (area under the curve=0.80).[66]

The ***Psy-Flex*** is a six-item self-reported questionnaire measuring psychological flexibility by rating on a 5-point Likert scale (5 – "very often" to 1 – "very rarely").[67] A higher score indicates a greater psychological flexibility. The Cronbach's α of the original version was 0.91 for the total sample and ranged from 0.78 to 0.97 for subsamples, representing a good internal consistency of the Psy-Flex in both clinical and non-clinical adults. The reliability and validity of the Chinese version of the Psy-Flex were assessed in a sample of 248 Chinese parents of children with autism. The results demonstrated adequate internal consistency (Cronbach's α = 0.84) and test–retest stability (weighted kappa = 0.88), along with a well-fitting one-factor structure for the Psy-Flex-C ($\chi^2/df = 1.62$, $p = 0.125$, RMSEA = 0.05, GFI = 0.99, CFI = 0.99, TLI = 0.98, SRMR = 0.02).[68]

The ***Parenting Sense of Competence Scale (PSOC)***[69] is a 17-item self-reported questionnaire assessing parenting competence by rating on a 6-point Likert scale (1 – "strongly disagree" to 6 – "strongly agree"). This instrument was categorised into two subscales of parenting, including the efficacy subscale (eight-item; test parents' perceived self-efficacy in the parenting role) and the satisfaction subscale (nine-item; examine parents' satisfaction and comfort with the parenting role). Higher scores indicate a higher sense of parenting competence in parenting. The Chinese version of the SDQ has been tested and validated in Chinese parents,

teachers, and students (aged 6–15 years), including those with and without ADHD. Internal consistency for most factors across the parent-, teacher-, and self-report versions was moderate to high (Cronbach's α = 0.55–0.86), with the exception of a lower α for the 'Peer Problems' factor in the self-report version (α = 0.40). Four-week test–retest reliability was satisfactory (intraclass correlations = 0.67–0.91).[70]

Secondary outcomes for children

The ***Strengths and Difficulties Questionnaire (SDQ)*** is a widely used 25-item parent-rated questionnaire to measure behaviour, emotion, and adjustment of children aged 2 to 17[71]. A total of five subscales were measured, including emotional symptoms, conduct problems, hyperactive-inattention, peer relationships, and prosocial behaviour. Each item is scored by the parent as follows: Never = 0, Somewhat True = 1, or Definitely True = 2. The scores for hyperactivity, emotional symptoms, conduct problems, and peer problems can be summed to generate a total difficulties score ranging from 0 to 40, whereas the prosocial score is not incorporated in the reverse direction into the total difficulties score. Higher scores in the total difficulties or subscales measuring child difficulties reflect greater difficulty levels, while higher scores in the prosocial behaviour subscale reflect greater child strengths. The Chinese version of the SDQ has been tested and validated in Chinese parents, teachers, and students (aged 6–15 years), including those with and without ADHD. Internal consistency for most factors across the parent-, teacher-, and self-report versions was moderate to high (Cronbach's α = 0.55–0.86), with the exception of a lower α for the 'Peer Problems' factor in the self-report

version (α = 0.40). Four-week test–retest reliability was satisfactory (intraclass correlations = 0.67–0.91).[72]

All the measurement tools are valid and reliable in both original English and translated Chinese versions and have been approved for use.

5.2.11 Data collection procedure

Primary and secondary outcomes will be assessed at baseline (T0), immediately post-intervention (T1), and six months post-intervention (T2). All quantitative data will be collected via an online questionnaire platform (WJX.com) with support from a trained research assistant (RA) who will be blinded to group allocation.

Baseline data collection. After obtaining written informed consent, baseline data—including demographics and all primary and secondary outcomes—will be collected. During baseline, an RA will provide in-person assistance to participants who experience any difficulty using the online platform.

Post-intervention data collection. The RA will contact and remind participants in both groups via WeChat, telephone, and/or email to complete post-intervention assessments at the designated time points. At T1, participants in the intervention group will complete questionnaires for all primary and secondary outcomes and an intervention satisfaction form within one week after completing all sessions. During the same period, participants in the control group will complete the same set of outcome questionnaires (without the satisfaction form). At T2 (six months post-intervention), the RA will contact/remind participants in both groups to complete follow-up assessments for all primary and secondary outcomes. At all three

time points, the RA will be available via WeChat and telephone to assist participants who encounter difficulties during data collection. The RA will review submitted questionnaires on the survey platform immediately after submission and will contact participants to complete any missing items when needed.

5.2.12 Data analysis

Data analysis will be conducted in R software (Version 4.4.1 for Mac). Descriptive statistics will summarize sample characteristics and study outcomes. Assumptions of normality and homogeneity will be assessed when examining baseline group differences. Outcome analyses will use generalized estimating equations (GEE) under the intention-to-treat (ITT) principle to compare differential changes between groups across time points. All randomized participants will be retained in the models in accordance with ITT. Missing data will be handled within the GEE framework using robust standard errors, providing valid population-averaged estimates under Missing Completely at Random (MCAR) or Missing at Random (MAR) assumptions. When the proportion of missing data exceeds 10%, multiple imputation will be performed.[73] If GEE results indicate significant effects, post hoc comparisons of estimated marginal means will be conducted to examine within-group and between-group differences for individual outcomes.

5.2.13 Treatment fidelity

The intervener received 157.5 hours of structured online ACT training developed by Dr. Steven C. Hayes and other ACT experts. The training can be accessed at https://www.praxiscet.com/. Additionally, the intervener completed a 5-hour parenting

program developed by the WHO (https://www.who.int/teams/mental-health-and-substance-use/treatment-care/who-caregivers-skills-training-for-families-of-children-with-developmental-delays-and-disorders). The intervener got certificates in both ACT-relevant skills and the WHO-CST. Prior to the pilot study, case scenario practices were conducted under the supervision of a senior ACT supervisor to ensure that the intervener is competent to carry out the intervention. The practitioner received regular supervision from a senior ACT supervisor to reduce intervention variability. All the intervention sessions will be audiotaped of the practitioner and participants after obtaining written consent from participants. Using a supervision form adapted from the ACT Fidelity Checklist and aligned to the session syllabus, the supervisor evaluated every activity in each session—including both ACT components and WHO-CST activities—for adherence, competence, and coverage. Fidelity feedback will provide after each review, and any deviations triggered targeted guidance and, when needed, additional practice. The supervision form was published in the Appendix Table 3 of https://doi.org/10.1177/13623613241311323. Supervisors will provide feedback to the practitioner after each rating. The intervener and supervisors will meet regularly to ensure the practitioner is following the protocol and is competent at giving the intervention. When necessary, remedial actions (e.g., additional training and practice) will be undertaken to ensure intervention fidelity. A face-to-face training program will be implemented for all RAs to ensure adherence to work ethics.

5.2.14 Ethical Considerations

The design and implementation of this study followed the Declaration of Helsinki, which is a statement of ethical principles for medical research involving human subjects. Ethical approval for this study was obtained from the Joint Chinese University of Hong Kong – New Territories East Cluster Clinical Research Ethics Committee (CREC Reference No.: 2023.590-T). Moreover, this study was registered with the Chinese Clinical Trial Registry (ChiCTR2400080472) before its commencement. Permission for conducting this study was also be obtained from the rehabilitation institutions. Ethical issues will be appropriately managed, including obtaining informed consent, ensuring anonymity and data confidentiality, and considering the potential benefits and risks of participation.

All eligible participants will be required to read and understand the participant information sheet and sign the consent form before any data collection. The information sheet will describe the study aims, data collection procedures, confidentiality protections, potential benefits and risks, voluntary participation, and the right to withdraw at any time. Participants in the control group will be permitted to seek help from other services. In accordance with the principle of beneficence, control-group participants will be offered the intervention, at their discretion, after completing the final data collection (six-month follow-up).

To ensure safe, stable, and smooth delivery of the final four sessions, the online videoconferencing platform Tencent Meeting (the largest such platform in China) will be used. A meeting password will be set, and only researchers and participants assigned to the relevant group will be able to join.

After completion of the final data collection (T2), participants will receive a small token of appreciation (valued at approximately 30 RMB) to acknowledge their time and contribution. These incentives will be clearly stated in the ethics application and explained in the informed consent.

Personally identifying data will be encoded to ensure anonymity. All electronic data will be stored in password-protected cloud storage (e.g., Google Drive). Only the research team will have access to the data. Personal information will be retained for six years after study completion and then destroyed.

5 References

1. American Psychiatric Association D, Association AP. *Diagnostic and statistical manual of mental disorders: DSM-5*. vol 5. American psychiatric association Washington, DC; 2013.
2. Zeidan J, Fombonne E, Scorah J, et al. Global prevalence of autism: A systematic review update. *Autism Research*. 2022;15(5):778-790. doi:https://doi.org/10.1002/aur.2696
3. Maenner MJ, Shaw KA, Bakian AV, et al. Prevalence and Characteristics of Autism Spectrum Disorder Among Children Aged 8 Years - Autism and Developmental Disabilities Monitoring Network, 11 Sites, United States, 2018. *MMWR Surveill Summ*. Dec 3 2021;70(11):1-16. doi:10.15585/mmwr.ss7011a1
4. Zhou H, Xu X, Yan WL, et al. Prevalence of Autism Spectrum Disorder in China: A Nationwide Multi-center Population-based Study Among Children Aged 6 to 12 Years. *Neurosci Bull*. Sep 2020;36(9):961-971. doi:10.1007/s12264-020-00530-6
5. Popow C, Ohmann S, Plener P. Practitioner's review: medication for children and adolescents with autism spectrum disorder (ASD) and comorbid conditions. *neuropsychiatrie*. 2021/09/01 2021;35(3):113-134. doi:10.1007/s40211-021-00395-9
6. Chiang H-M, Wineman I. Factors associated with quality of life in individuals with autism spectrum disorders: A review of literature. *Research in autism spectrum disorders*. 2014;8(8):974-986.
7. Lockington DC, Gullon-Scott F. The Lived Experiences of Autistic Mothers: A Systematic Review and Thematic Synthesis of Qualitative Evidence. *Autism & Developmental Language Impairments*. 2025;10:23969415251343850.
8. Ng CSM, Fang Y, Wang Z, Zhang M. Potential Factors of Parenting Stress in Chinese Parents of Children With Autism Spectrum Disorder: A Systematic Review. *Focus on Autism and Other Developmental Disabilities*. 2021;36(4):237-248. doi:10.1177/10883576211012599
9. Kurzrok J, McBride E, Grossman RB. Autism-specific parenting self-efficacy: An examination of the role of parent-reported intervention involvement, satisfaction with intervention-related training, and caregiver burden. *Autism*. Jul 2021;25(5):1395-1408. doi:10.1177/1362361321990931
10. Drouillard BE. *Supporting Treatment Selection in Parents of Children with Autism Spectrum Disorder: An Educational Workshop with Acceptance and Commitment Training*. University of Windsor (Canada); 2019.
11. Gatzoyia D, Kotsis K, Koullourou I, et al. The association of illness perceptions with depressive symptoms and general psychological distress in parents of an offspring with autism spectrum disorder. *Disability and Health Journal*. 2014/04/01/ 2014;7(2):173-180. doi:https://doi.org/10.1016/j.dhjo.2013.10.008
12. van der Lubbe A, Swaab H, Vermeiren RRJM, Ester WA. Stress, Eating Behavior and Adverse Health in Parents of Young Children with Autism Spectrum Disorder. *J Autism Dev Disord*. 2022/11/25 2022;doi:10.1007/s10803-022-05825-3

13. Lee GK, Lopata C, Volker MA, et al. Health-related quality of life of parents of children with high-functioning autism spectrum disorders. *Focus on autism and other developmental disabilities*. 2009;24(4):227-239.
14. Moreno Méndez JH, Giraldo Jiménez CS, Avendaño Prieto BL. Psychological Inflexibility and Adherence to the Therapy among Parents of Autistic Children. *Avances en Psicología Latinoamericana*. 2020;38(2):146-158.
15. Estes A, Munson J, Dawson G, Koehler E, Zhou X-H, Abbott R. Parenting stress and psychological functioning among mothers of preschool children with autism and developmental delay. *Autism*. 2009;13(4):375-387.
16. Operto F, G P, C S, et al. Adaptive Behavior, Emotional/Behavioral Problems and Parental Stress in Children With Autism Spectrum Disorder. Article. *Front Neurosci Journal Translated Name Frontiers in Neuroscience Keyword Heading adaptive functioning autism spectrum disorder emotional/behavioral problems neurodevelopmental disorders parental stress*. 25 Nov 2021;15:no pagination. doi:https://dx.doi.org/10.3389/fnins.2021.751465
17. Chen Y, Cheng T, Lv F. Sense of Parenting Efficacy, Perceived Family Interactions, and Parenting Stress Among Mothers of Children With Autistic Spectrum Disorders. *Front Psychol*. 2022;13:878158. doi:10.3389/fpsyg.2022.878158
18. Althoff CE, Dammann CP, Hope SJ, Ausderau KK. Parent-Mediated Interventions for Children With Autism Spectrum Disorder: A Systematic Review. *Am J Occup Ther*. May/Jun 2019;73(3):7303205010p1-7303205010p13. doi:10.5014/ajot.2019.030015
19. Gould ER, Tarbox J, Coyne L. Evaluating the effects of Acceptance and Commitment Training on the overt behavior of parents of children with autism. *Journal of Contextual Behavioral Science*. 2018/01/01/ 2018;7:81-88. doi:https://doi.org/10.1016/j.jcbs.2017.06.003
20. Rutherford M, Singh-Roy A, Rush R, McCartney D, O'Hare A, Forsyth K. Parent focused interventions for older children or adults with ASD and parent wellbeing outcomes: A systematic review with meta-analysis. *Research in Autism Spectrum Disorders*. 2019/12/01/ 2019;68:101450. doi:https://doi.org/10.1016/j.rasd.2019.101450
21. MacKenzie KT, Eack SM. Interventions to Improve Outcomes for Parents of Children with Autism Spectrum Disorder: A Meta-Analysis. *J Autism Dev Disord*. Jul 2022;52(7):2859-2883. doi:10.1007/s10803-021-05164-9
22. Li SN, Chien WT, Lam SKK, Chen ZY, Ma X. Effectiveness of parent-focused interventions for improving the mental health of parents and their children with autism spectrum disorder: A systematic review and meta-analysis. *Research in Autism Spectrum Disorders*. 2024/06/01/ 2024;114:102389. doi:https://doi.org/10.1016/j.rasd.2024.102389
23. Hayes SC. Acceptance and commitment therapy, relational frame theory, and the third wave of behavioral and cognitive therapies. *Behavior Therapy*. 2004/09/01/ 2004;35(4):639-665. doi:https://doi.org/10.1016/S0005-7894(04)80013-3
24. Çiçek Gümüş E, Öncel SJCP. Effects of Acceptance and Commitment Therapy-based interventions on the mental states of parents with special needs children: Randomized controlled trial. 2022:1-14.

25. Marino F, Failla C, Chilà P, et al. The Effect of Acceptance and Commitment Therapy for Improving Psychological Well-Being in Parents of Individuals with Autism Spectrum Disorders: A Randomized Controlled Trial. 2021;11(7):880.
26. Hahs AD, Dixon MR, Paliliunas DJJoCBS. Randomized controlled trial of a brief acceptance and commitment training for parents of individuals diagnosed with autism spectrum disorders. 2019;12:154-159.
27. Zody MC. *Acceptance and commitment therapy (ACT) as a one-day workshop for parents of children with an autism spectrum disorder*. Psy.D. The Wright Institute; 2017.
28. Singh NN, Lancioni GE, Medvedev ON, Hwang Y-S, Myers RE. A component analysis of the mindfulness-based positive behavior support (MBPBS) program for mindful parenting by mothers of children with autism spectrum disorder. *Mindfulness*. 2021;12:463-475.
29. Prata J, Lawson W, Coelho R. Parent training for parents of children on the autism spectrum: A review. *Int J Clin Neurosci Ment Health*. 2018;5:3.
30. Salomone E, Ferrante C, Salandin A, et al. Acceptability and feasibility of the World Health Organization's Caregiver Skills Training implemented in the Italian National Health System. *Autism : the international journal of research and practice*. May 2022;26(4):859-874. doi:10.1177/13623613211035228
31. Flujas-Contreras JM, García-Palacios A, Castilla D, Gómez I. Internet-based versus face-to-face Acceptance and Commitment Therapy for parental psychological flexibility. *Current Psychology*. 2023/08/25 2023;doi:10.1007/s12144-023-05052-8
32. Corralejo SM, Domenech Rodríguez MM. Technology in parenting programs: A systematic review of existing interventions. *Journal of Child and Family Studies*. 2018;27:2717-2731.
33. Hermaszewska S, Sin J. End-user perspectives on the development of an online intervention for parents of children on the autism spectrum. *Autism*. 2021;25(5):1234-1245.
34. Foody M, Barnes-Holmes Y, Barnes-Holmes D, et al. RFT for clinical use: The example of metaphor. *Journal of Contextual Behavioral Science*. 2014;3(4):305-313.
35. Brown FL, Whittingham K, Boyd RN, McKinlay L, Sofronoff KJBr, therapy. Does Stepping Stones Triple P plus Acceptance and Commitment Therapy improve parent, couple, and family adjustment following paediatric acquired brain injury? A randomised controlled trial. 2015;73:58-66.
36. Perry A. A model of stress in families of children with developmental disabilities: Clinical and research applications. *Journal on developmental disabilities*. 2004;11(1):1-16.
37. Enea V, Rusu D. Raising a child with autism spectrum disorder: A systematic review of the literature investigating parenting stress. *Journal of Mental Health Research in Intellectual Disabilities*. 2020;13(4):283-321.
38. Fonseca A, Moreira H, Canavarro MC. Uncovering the links between parenting stress and parenting styles: The role of psychological flexibility within parenting and global psychological flexibility. *Journal of contextual behavioral science*. 2020;18:59-67.

39. Huang M, Zhou Z. Perceived self-efficacy, cultural values, and coping styles among Chinese families of children with autism. *International Journal of School & Educational Psychology*. 2016;4(2):61-70.
40. *Diagnostic and statistical manual of mental disorders: DSM-5™, 5th ed.* Diagnostic and statistical manual of mental disorders: DSM-5™, 5th ed. American Psychiatric Publishing, Inc.; 2013:xliv, 947-xliv, 947.
41. Erikson EH. *Identity youth and crisis*. WW Norton & company; 1968.
42. Emerson E, Kiernan C, Alborz A, et al. The prevalence of challenging behaviors: A total population study. *Research in developmental disabilities*. 2001;22(1):77-93.
43. Nieto C, López B, Gandía H. Relationships between atypical sensory processing patterns, maladaptive behaviour and maternal stress in Spanish children with autism spectrum disorder. *J Intellect Disabil Res*. Dec 2017;61(12):1140-1150. doi:10.1111/jir.12435
44. Samadi H, Samadi SA. Understanding Different Aspects of Caregiving for Individuals with Autism Spectrum Disorders (ASDs) a Narrative Review of the Literature. *Brain Sci*. Aug 14 2020;10(8)doi:10.3390/brainsci10080557
45. Boutron I, Altman DG, Moher D, Schulz KF, Ravaud P. CONSORT Statement for Randomized Trials of Nonpharmacologic Treatments: A 2017 Update and a CONSORT Extension for Nonpharmacologic Trial Abstracts. *Ann Intern Med*. Jul 4 2017;167(1):40-47. doi:10.7326/m17-0046
46. Ministry of Education of the People's Republic of China. Guidelines for quality evaluation of special education. [特殊教育办学质量评价指南]. Accessed 22th September, 2023. http://www.moe.gov.cn/jyb_xwfb/gzdt_gzdt/s5987/202211/t20221107_976412.html
47. Federation. SDP. Notice from the Shenzhen Disabled Persons' Federation on the Issuance of the 'Implementation Measures for Rehabilitation Assistance for Disabled Children in Shenzhen. 2024. https://www.sz.gov.cn/zfgb/2022/gb1254/content/post_10026447.html
48. Marino F, Failla C, Chilà P, et al. The Effect of Acceptance and Commitment Therapy for Improving Psychological Well-Being in Parents of Individuals with Autism Spectrum Disorders: A Randomized Controlled Trial. *Brain Sci*. Jun 30 2021;11(7)doi:10.3390/brainsci11070880
49. Çiçek Gümüş E, Öncel S. Effects of Acceptance and Commitment Therapy-based interventions on the mental states of parents with special needs children: Randomized controlled trial. *Current Psychology*. 2023/08/01 2023;42(23):19429-19442. doi:10.1007/s12144-022-03760-1
50. Fewtrell MS, Kennedy K, Singhal A, et al. How much loss to follow-up is acceptable in long-term randomised trials and prospective studies? *Archives of disease in childhood*. 2008;93(6):458-461.
51. Dykens EM, Fisher MH, Taylor JL, Lambert W, Miodrag N. Reducing distress in mothers of children with autism and other disabilities: A randomized trial. Specialized Interventions 3350. *Pediatrics*. 2014;134(2):e454-e463. doi:https://dx.doi.org/10.1542/peds.2013-3164

52. Ede MO, Anyanwu JI, Onuigbo LN, et al. Rational emotive family health therapy for reducing parenting stress in families of children with autism spectrum disorders: A group randomized control study. Group & Family Therapy 3313. *Journal of Rational-Emotive & Cognitive-Behavior Therapy*. 2020;38(2):243-271. Journal of Rational-Emotive Therapy, Rational Living. doi:https://dx.doi.org/10.1007/s10942-020-00342-7
53. Tonge B, Brereton A, Kiomall M, Mackinnon A, Rinehart NJ. A randomised group comparison controlled trial of 'preschoolers with autism': a parent education and skills training intervention for young children with autistic disorder. *Autism : the international journal of research and practice*. Feb 2014;18(2):166-77. doi:10.1177/1362361312458186
54. Tonge B, Brereton A, Kiomall M, et al. Effects on parental mental health of an education and skills training program for parents of young children with autism: A randomized controlled trial. 2006;45(5):561-569.
55. Weitlauf AS, Broderick N, Stainbrook JA, et al. Mindfulness-based stress reduction for parents implementing early intervention for autism: An RCT. 2020;145(Supplement_1):S81-S92.
56. Weitlauf AS, Broderick N, Alacia Stainbrook J, et al. A Longitudinal RCT of P-ESDM With and Without Parental Mindfulness Based Stress Reduction: Impact on Child Outcomes. *Journal of Autism and Developmental Disorders*. 2022;
57. Faul F, Erdfelder E, Lang A-G, Buchner A. G*Power 3: A flexible statistical power analysis program for the social, behavioral, and biomedical sciences. *Behavior Research Methods*. 2007/05/01 2007;39(2):175-191. doi:10.3758/BF03193146
58. Altman DG, Bland JM. How to randomise. *Bmj*. Sep 11 1999;319(7211):703-4. doi:10.1136/bmj.319.7211.703
59. Harris R, Hayes SC. *ACT Made Simple: An Easy-To-Read Primer on Acceptance and Commitment Therapy*. New Harbinger Publications; 2019.
60. Harris R. *The Happiness Trap: Stop Struggling, Start Living*. Exisle Publishing; 2007.
61. Luo J, Wang MC, Gao Y, et al. Refining the Parenting Stress Index-Short Form (PSI-SF) in Chinese Parents. *Assessment*. Mar 2021;28(2):551-566. doi:10.1177/1073191119847757
62. Abidin R. The Parenting Stress Index-Short Form. *Charlottesville, VA: Pediatric Psychology Press*. 1990;
63. Kroenke K, Spitzer RL, Williams JBW. The PHQ-9: Validity of a brief depression severity measure. Article. *Journal of General Internal Medicine*. 2001;16(9):606-613. doi:10.1046/j.1525-1497.2001.016009606.x
64. Wang W, Bian Q, Zhao Y, et al. Reliability and validity of the Chinese version of the Patient Health Questionnaire (PHQ-9) in the general population. *Gen Hosp Psychiatry*. Sep-Oct 2014;36(5):539-44. doi:10.1016/j.genhosppsych.2014.05.021
65. Spitzer RL, Kroenke K, Williams JB, Löwe B. A brief measure for assessing generalized anxiety disorder: the GAD-7. *Archives of internal medicine*. 2006;166(10):1092-1097.

66. Zeng Q-Z, He Y-L, Liu H, et al. Reliability and validity of Chinese version of the Generalized Anxiety Disorder 7-item (GAD-7) scale in screening anxiety disorders in outpatients from traditional Chinese internal department. *Chinese Mental Health Journal*. 2013;27(3):163-168.
67. Gloster AT, Block VJ, Klotsche J, et al. Psy-Flex: A contextually sensitive measure of psychological flexibility. *Journal of Contextual Behavioral Science*. 2021;22:13-23.
68. Li SN, Chien WT, Lam SKK, Chong YY, Gloster AT. Psychometric Properties of the Chinese Version of the Psy-Flex Among Parents of Children with Autism Spectrum Disorder. *J Autism Dev Disord*. Oct 27 2024;doi:10.1007/s10803-024-06610-0
69. Johnston C, Mash EJ. A measure of parenting satisfaction and efficacy. *Journal of clinical child psychology*. 1989;18(2):167-175.
70. Li XY, Mao KN, Mi XY, et al. [Reliability and validity of the Chinese version of parenting sense of competence scale in mothers of preschool children]. *Beijing Da Xue Xue Bao Yi Xue Ban*. 2021/06// 2021;53(3):479-484. doi:10.19723/j.issn.1671-167x.2021.03.007
71. Goodman R, Ford T, Simmons H, Gatward R, Meltzer H. Using the Strengths and Difficulties Questionnaire (SDQ) to screen for child psychiatric disorders in a community sample. *Br J Psychiatry*. Dec 2000;177:534-9. doi:10.1192/bjp.177.6.534
72. Liu SK, Chien YL, Shang CY, Lin CH, Liu YC, Gau SS. Psychometric properties of the Chinese version of Strength and Difficulties Questionnaire. *Compr Psychiatry*. Aug 2013;54(6):720-30. doi:10.1016/j.comppsych.2013.01.002
73. Madley-Dowd P, Hughes R, Tilling K, Heron J. The proportion of missing data should not be used to guide decisions on multiple imputation. *Journal of clinical epidemiology*. 2019;110:63-73.

Table 1. Session-by-session outline of the ACT-based parenting programme

**Theme [Time & Duration]; Delivery formats**	**ACT/parenting skills training core principle(s)**	**Aim**	**Key activities**	**The adaptation and integration of ACT/WHO-CST in the context of parenting an autistic child**
Session 1: Introduction and engaging [Week 1 & 120 minus]; Face-to-face	ACT: Contact to present moment, compassion, creative hopelessness, and acceptance<br>PST: Autism-related education	Help participants experience mindfulness exercises for the first time and quickly focus on the present moment in the classroom	A quick mindfulness exercise	**ACT modification**: The example of experiential avoidance provided in the ACT manual (Harris, 2019) is a general scenario and not relevant to the context of parenting an autistic child. Therefore, to help parents recognise and identify their experiential avoidance thoughts and subsequent experiential avoidant behaviours in caregiving, we have provided various examples based on different forms of experiential avoidance in parenting context. For example, a parent and child may agree to buy only one toy before going to the supermarket. If the child sees multiple toys and insists on more, the parent may refuse, leading to a tantrum. To avoid discomfort—like the disapproving gaze of others—the parent might eventually give in. This pattern of excessive concession can lead to inconsistent parenting and reinforce the child's problematic behaviours, causing the child to rely on these behaviours to communicate their needs. Moreover, adapting the Chinese idiom "Grasshoppers tied to the same rope" ("一根繩上的蚂蚱" in Mandarin Chinese) in place of the original metaphor of 'Two mountains' in the ACT manual (Harris, 2019) can more effectively clarify the therapeutic relationship and establish rapport for Chinese population. This idiom underscores that both the therapist and the participants are part of a shared community of interests, united by a common goal: to assist parents of autistic children in better managing caregiving challenges.
		To introduce the programme and group members to the group and build peer and therapeutic relationships	Welcome for participants and introduction for the programme<br>Self-introduction among group members<br>"Grasshoppers tied to the same rope" metaphor	
		Give a whole awareness of autism	Education on the topic of "Overview" of autism	
		Identify participants' parenting	Identify challenges for parenting an autistic child	

Theme [Time & Duration]; Delivery formats	ACT/parenting skills training core principle(s)	Aim	Key activities	The adaptation and integration of ACT/WHO-CST in the context of parenting an autistic child
		challenges, ineffective parental stress coping strategies, experiential avoidance behaviours, and their related consequences by using creative hopelessness technique.	Identify participants' ineffective coping strategies<br>Introduce experiential avoidance<br>The "Tug of war with a Monster" and "white bear" metaphors<br>Turning pain into resources	**WHO-CST modification**: The WHO-CST manual provides a brief overview of developmental delays and disabilities. Therefore, based on the "Chinese Guideline for Diagnosis, Treatment, and Rehabilitation of Autism in Children" (Ministry of Health of the People's Republic of China, 2010), we have revised this section to focus specifically on a detailed overview of autism. This overview includes the characteristics of autism, epidemiology, comorbidities, risk factors, and interventions for autistic children.<br>**Integration of ACT and parenting skill training**: In the educational activities focused on the "Overview of Autism", intervener enumerated various challenges faced by autistic children, as well as the experiential avoidance behaviours that parents exhibited when confronted with these problems. Our goal was to help parents recognise their own patterns of experiential avoidance, along with the behaviours and impacts associated with their parenting and self-care journey. We used the experiential exercise of "Tug of War with a Monster" and the "White Bear" metaphor to assist parents in practising acceptance of their thoughts and feelings regarding parenting. This process involved acknowledging their emotions without judgment, thereby encouraging them to engage with their experiences related to raising an autistic child rather than avoiding them. Moreover, the 5-minute mindfulness parenting practice assigned as homework for this session allows parents to focus on their breath and observe their thoughts without judgment during stressful events in parenting, helping to cultivate a calm mindset
		Homework assignment	Setting homework: A 5-minute quick mindfulness parenting exercise	
		Recap	Wrap-up and group discussion	

Theme [Time & Duration]; Delivery formats	ACT/parenting skills training core principle(s)	Aim	Key activities	The adaptation and integration of ACT/WHO-CST in the context of parenting an autistic child
				in parenting. The guiding mindfulness script was adapted from a general context to a parenting-related context.
Session 2: Acceptance and values identification [Week 2 & 120 minus]; Face-to-face	ACT: Contact to present moment, acceptance, values, and committed action<br>PST: Implementing actions based on their parenting values	Help parents explore their value and help them realise the relationship between value and pain<br>To identify their attitudes, values, and motivations for parenting and personal development (e.g., self-care)<br>Homework review and assignment	"Two Hands" experiential exercise and group discussion<br>"The 80th birthday party" mindfulness experiential exercise and group discussion<br>Introduce values using "Journey to the West" metaphor<br>Identify your values: The Bull's Eye worksheet<br>Commitment action worksheet<br>Review of home practice of mindfulness parenting exercise<br>Setting homework: commitment actions	**ACT modification**: In Chinese culture, discussing death is often considered a taboo topic (Xu, 2007). This avoidance stems from a profound understanding and respect for life and death; directly mentioning death is viewed as inauspicious and may invite misfortune (Lei et al., 2022). Therefore, we adapted 'The Video of Your Mistakenly Held Funeral' mindfulness exercise in the ACT manual (Harris, 2019) to 'The 80th Birthday Party', facilitating a more celebratory context for parents of autistic children to explore their values. Moreover, the mindfulness script of 'The 80th Birthday Party' was modified from a general script to better fit the parenting context, assisting parents in identifying their core parenting values. Moreover, Journey to the West ("西遊記" in Mandarin Chinese) is one of the four great classical novels of Chinese literature, telling the mythological story of a monk who embarks on a journey to the West to obtain sacred scriptures, facing 9,981 trials along the way before finally achieving his goal. This story is widely known and has been adapted into animated series that are highly praised and loved by children in China. In our intervention protocol, this metaphor was applied to help participants understand the distinction between values and goals. In addition, unlike the ACT manual (Harris, 2019), which offers a single general worksheet for personal values, this programme adapted the Bull's Eye worksheets into two versions to help parents separately identify their personal and parenting values.

Theme [Time & Duration]; Delivery formats	ACT/parenting skills training core principle(s)	Aim	Key activities	The adaptation and integration of ACT/WHO-CST in the context of parenting an autistic child
		Recap	Wrap-up and group discussion	Value clarification can be facilitated through an exploration exercise in which parents identify their own values related to parenting (e.g., being patient, supportive, or present in their relationship with their autistic child), rather than focusing on aspirations for others (e.g., their child's future independence or happiness). This process may culminate in a vision board or a written commitment statement that serves as a personal reminder of their chosen parenting values during challenging times. After clarifying their parenting values, parents can set specific, measurable goals related to their child's development. This process involves breaking these goals down into small, actionable steps. For example, they might commit to spending 10 minutes daily on turn-taking games. By focusing on these manageable tasks, parents can create a structured approach to support their child's growth while aligning their actions with their core values. This not only enhances the child's development but also fosters a sense of accomplishment for the parents.<br>**WHO-CST modification**: None.<br>**Integration of ACT and parenting skill training**: By employing ACT techniques such as values clarification and developing weekly parenting support actions based on parenting values, parents can apply the parenting skills learned in the classroom to their daily routines. This approach enables parents to set specific, measurable goals related to their child's development, breaking these goals down into small, actionable steps, such as dedicating 10 minutes each day to turn-taking games.

Theme [Time & Duration]; Delivery formats	ACT/parenting skills training core principle(s)	Aim	Key activities	The adaptation and integration of ACT/WHO-CST in the context of parenting an autistic child
Session 3: Mindful parenting and parent-child interaction (1) [Week 3 & 120 minus]; Face-to-face	ACT: Contact to present moment, cognitive defusion, committed action<br>PST: Engaging autistic children in interactive activities	To introduce the concept of cognitive fusion for participants, help they to see the true nature of cognitions, and teach they how to use strategies to defusion	"Time Travel" mindfulness experiential exercise, group discussion, and followed by the "Passengers on the bus" metaphor<br>"Lemon" experiential exercise, introduce cognitive fusion, and practice defusion strategies	**ACT modification**: The examples of cognitive fusion and the techniques for cognitive defusion in the ACT manual (Harris, 2019) are general and not relevant for parenting an autistic child. For each instance of cognitive fusion, we adapted specific examples relevant to the parenting context. For example, we teach parents cognitive defusion techniques to create distance from their thoughts, particularly regarding the challenges they face in parenting. When autistic children exhibit challenging behaviours in public, parents can reframe their thoughts by saying, 'I am having the thought that I am a bad parent because I cannot manage my child's behaviours,' instead of simply thinking, 'I am a bad parent because I cannot manage my child's behaviours.' This shift helps them recognise thoughts as transient rather than definitive.
		To help participants identify their children's interaction styles and learn interaction skills<br>Helping children to share engagement in home routine	Identify your child's level of engagement<br>Tips for sharing engagement with your child<br>Helping children to share engagement in home routines	**WHO-CST modification**: The WHO-CST manual introduces different levels of assistance that parents can provide to their children with developmental delays and disabilities: Level 1 involves only verbal assistance; Level 2 includes verbal and physical assistance at the beginning and end of the activity; and Level 3 entails using verbal and physical assistance throughout the entire activity. It also provides a general example, such as how to help a child button their clothes. To help participants better understand how to apply these varying levels of support in daily caregiving, our protocol includes examples specifically related to parenting an autistic child. For example, parents can schedule regular sensory breaks

Theme [Time & Duration]; Delivery formats	ACT/parenting skills training core principle(s)	Aim	Key activities	The adaptation and integration of ACT/WHO-CST in the context of parenting an autistic child
			Identify your level of help	throughout the day, where the child participates in activities like jumping on a trampoline or playing with water. This helps the child manage sensory overload and promotes overall well-being. At Level 1, parents use only verbal assistance, guiding the child to engage in activities like jumping on a trampoline or using a stress ball, allowing for independent choice. At Level 2, parents combine verbal and physical support at the beginning and end of the break; they might demonstrate a sensory activity while explaining it and help the child transition back to the main activity afterward. At Level 3, parents provide full support throughout the break, actively participating by guiding the child through each step, holding their hand, and demonstrating how to engage with sensory materials. By adjusting the level of support, parents can help their children effectively manage sensory overload and promote emotional regulation.<br>**Integration of ACT and parenting skill training**: When parents formulate and implement daily family routines, they may make decisions detrimental to their child's upbringing and development due to cognitive fusion. For instance, parents may excessively worry that their child will exhibit problematic behaviours in public settings, leading them to decide to reduce outings with their child. However, the development of a child's social and language skills requires interaction with external environments and individuals. Therefore, this lesson aims to highlight common instances of cognitive fusion experienced by parents of autistic children, along with the resulting behaviours and parenting impacts, to assist parents in more
		Homework review and assignment	Review of homework practice and noticing barriers<br>Setting homework: 1) Home routines work sheet; 2) commitment actions	
		Recap	Wrap-up and group discussion	

Theme [Time & Duration]; Delivery formats	ACT/parenting skills training core principle(s)	Aim	Key activities	The adaptation and integration of ACT/WHO-CST in the context of parenting an autistic child
				effectively designing daily family activities that are most suitable for their child. The guide script of the homework mindfulness exercise was modified to fit the parenting context so that parents can practice mindfulness in daily caregiving.
Session 4: Mindful parenting and parent-child interaction (2) [Week 4 & 120 minus]; Face-to-face	ACT: Contact to present moment, commitment action<br>PST: Promoting children to share engagement in home and play routine	To introduce the concept of contact to present moment, improve the awareness for participants, and help participants practice these strategies in their caregiving<br>Helping children to share engagement in play routine	Contact to present moment mindfulness exercises (mindful eating a raisin) and group discussions<br>Four types of children's games and introduce steps/tips for engaging children in family daily play routine<br>Specific examples of different types of children's games	**ACT modification**: After guiding parents through the mindful eating exercise, the discussion section was adapted from a general recap (e.g., 'Are you fully present in your daily life?') to a parenting-specific context. This adaptation facilitates participants in sharing their feelings about the practice and prompts them to reflect on their ability to fully engage in interactions, conversations, and play with their children. This awareness can help them notice their children's responses and needs that they might typically overlook.<br>**WHO-CST modification**: The WHO-CST manual only outlines three specific games in a step-by-step guide for children with developmental delays and disabilities: building with blocks, playing with cars, and making cakes with playdough. Considering that play is a primary mode of interaction between children and parents, we adapted specific games in the book '*Play and Rehabilitation for Children with Autism*' (Wang, 2018). These games aim to enhance children's language skills, social interactions, emotional management and expression, sensory development, the cultivation of positive behaviours, and cognitive abilities.<br>**Integration of ACT and parenting skill training**: Encourage parents to practice mindful parenting by being fully engaged during daily activities with their child,

Theme [Time & Duration]; Delivery formats	ACT/parenting skills training core principle(s)	Aim	Key activities	The adaptation and integration of ACT/WHO-CST in the context of parenting an autistic child
			Challenges parents may encounter in interactive activities/games and ways to address them.	such as during playtime or mealtime. This involves putting away distractions, such as phones, and fully focusing on the moment. Moreover, parents are encouraged to engage their senses to bring their attention to the present, especially during parenting. For example, they can focus on the sounds in their environment, such as their children's laughter, the texture of toys, or their autistic children's expressions and body movements. This sensory awareness helps parents remain grounded and fully engaged with their children, enhancing their connection and responsiveness in everyday interactions. By practising this mindfulness technique, parents can cultivate a deeper appreciation for the moments they share with their children. The guide script of the homework mindfulness exercise was modified to fit the parenting context so that parents can practice mindfulness in daily caregiving.
		Homework review and assignment	Review of homework practice and noticing barriers<br>Setting homework: 1) A mindfulness parenting exercise; 2) commitment actions	
		Recap	Wrap-up and group discussion	
Session 5: Understanding and promoting communication [Week 5 & 120 minus]; Online	ACT: Contact to present moment, self-as-content, commitment action	To introduce the concept of self-as-content and help participants enhance defusion, especially from the conceptualised self	Self-as-content introduction: "Notice your noticing" mindfulness experiential exercise and "The Sky and the Weather" and "Stage show" metaphors	**ACT modification**: The script for the "Notice Your Noticing" mindfulness practice was designed to equip parents with ACT techniques, specifically the concept of self-as-context, for use in daily caregiving. We adapted the guiding scripts of the mindfulness exercise from a general script (e.g., recalling a painful life event) to the parenting context by encouraging parents to recall a specific event from the past week or month that caused them significant emotional distress while raising their autistic children, thereby creating distance from those thoughts and

Theme [Time & Duration]; Delivery formats	ACT/parenting skills training core principle(s)	Aim	Key activities	The adaptation and integration of ACT/WHO-CST in the context of parenting an autistic child
	PST: Improving children's communication skills	Help participants learn and apply tips for advancing their child's communication skills during play and home routines	Identify your child's way to communication<br>Tips for understanding and promoting communication<br>Children's sharing and requirement<br>Four steps before bedtime: Review today and preview tomorrow<br>Tips for using picture books to help children enhance their language skills	experiences. Based on this event, we facilitated a self-as-context mindfulness practice for the participants. This process encourages them to recognise that they can manage their emotions, particularly in challenging situations. In addition, it helps parents identify their core values beyond their role as caregivers during that event. By reflecting on these values, parents can gain clarity and perspective, fostering a deeper understanding of themselves and their parenting journey.<br>**WHO-CST modification**: Specific activities and tips, including 'Four Steps Before Bedtime: Review Today and Preview Tomorrow' and 'Tips for Using Picture Books to Help Children Enhance Their Language Skills,' were added to assist parents in learning applicable and specific methods to promote communication with their autistic children. These two activities were recommended by an expert from our panel and are detailed in her book for parents of autistic children (Chang, 2023). These activities have been widely used by the parents she has assisted and have proven beneficial in promoting children's interactive communication. The WHO-CST manual also provides similar guidance for facilitating children's interactive communication; however, it does not offer specific practice examples. Consequently, we have adapted these two activities for this session, following the introduction of the parenting skills derived from WHO-CST. This adaptation aims to equip parents with more concrete steps and strategies for practical implementation.
		Homework review and assignment	Review of homework practice and noticing barriers<br>Setting homework: 1) Communication and noticing; 2) commitment actions	

Theme [Time & Duration]; Delivery formats	ACT/parenting skills training core principle(s)	Aim	Key activities	The adaptation and integration of ACT/WHO-CST in the context of parenting an autistic child
		Recap	Wrap-up and group discussion	**Integration of ACT and parenting skill training**: Incorporating "self-as-context" mindfulness exercises encourages parents to observe their thoughts and emotions during interactions and communication with their child, fostering non-judgmental awareness. This practice enables parents to effectively regulate their emotions and sensitively discern their child's potential needs through non-verbal cues, such as facial expressions and body language, particularly in children with limited language skills. Moreover, it helped parents seize appropriate opportunities to apply the parenting techniques learned in the classroom during interactions with their child, thereby fostering the child's language development.
Session 6: Challenging behaviour understanding [Week 6 & 120 minus]; Online	ACT: Contact to present moment, compassion, commitment action<br>PST: Identifying and understanding children's challenging behaviour	To introduce the concept of compassion to participants and cultivate compassion for themselves<br><br>To introduce the ways of self-care to participants	"A suffering friend" Metaphor<br>"Loving-Kindness Body Scan" mindfulness experiential exercise and group discussion<br>Introduce self-compassion<br>The ways to conduct self-care<br><br>Behaviour Thermometer	**ACT modification**: We adapted the guiding scripts of the "Loving-Kindness Body Scan" mindfulness exercise from a general context to the parenting context by encouraging parents to recognise their efforts in raising their children. For example, we modified the phrase 'Thank your feet for taking you so far' from the ACT manual to 'Thank your feet for their selfless dedication, allowing you to accompany your child for therapeutic interventions and outdoor play'.<br>**WHO-CST modification**: Based on the self-care strategies outlined in the WHO-CST, such as healthy eating, adequate sleep, and exercise, our intervention protocol adopted a more scientific and detailed explanation of various self-care practices for participants, grounded in guides for dietary nutrition (Chinese Society of Nutrition, 2022), sleep (Liu & Zhang, 2017), and exercise (General Administration of Sport of China, 2017). This includes information on dietary

**Theme [Time & Duration]; Delivery formats**	**ACT/parenting skills training core principle(s)**	**Aim**	**Key activities**	**The adaptation and integration of ACT/WHO-CST in the context of parenting an autistic child**
		Help participants identify their children's behavioural and emotional problems and their children's underlying needs	The potential reasons cause your child's challenging behaviours<br>The four goals for responding to challenging behaviour	nutritional intake and sources of nutritious foods, definitions of adequate sleep along with methods for falling asleep quickly, different exercise modalities categorised by intensity, and important considerations before and after exercise, among other topics.<br>**Integration of ACT and parenting skill training**: Parents recognised the importance of self-compassion and self-care through the "Loving-Kindness Body Scan" mindfulness exercise. This understanding enabled them to actively practice the self-care techniques learned in the classroom in their daily lives, ultimately enhancing their overall well-being and better supporting their children with autism. Building on the foundation of self-compassion, this session emphasises that children's emotional and behavioural problems often arise from unmet needs and highlights the potential reasons underlying various emotional and behavioural problems. By integrating compassion with parenting skills, a supportive and compassionate environment is fostered, where children's mistakes and their emotional and behavioural problems are seen as opportunities for growth rather than failures.
		Homework review and assignment	Review of home practice of valued action and noticing barriers<br>Setting homework: 1) Functional analysis diary; 2) commitment actions	
		Recap	Wrap-up and group discussion	
Session 7: Challenging behaviour management	ACT: Contact to present moment, compassion,	Help participants cultivate compassion for their autistic children	"Compassion for my autistic child" mindfulness exercise and group discussion	**ACT modification**: We adapted the guiding scripts of the mindfulness exercise from a general script (e.g., compassion for someone I love) to a specific parenting context for their autistic children by encouraging parents to recognise the

Theme [Time & Duration]; Delivery formats	ACT/parenting skills training core principle(s)	Aim	Key activities	The adaptation and integration of ACT/WHO-CST in the context of parenting an autistic child
[Week 7 & 120 minus]; Online	commitment action<br>PST: Challenging behaviour management	Help participants to learn how to deal with their children's behavioural and emotional problems<br>To help participants overcome affiliate stigma<br>Homework review and assignment<br>Recap	Tips to help children stay calm and cool<br>Steps to deal with your child's challenging behaviours<br>Introduce affiliate stigma<br>Review of home practice of valued action and noticing barriers<br>Setting homework: 1) Say Thanks to yourself and people you love; 2) commitment actions<br>Wrap-up and group discussion	challenges faced by their children and to convey compassion and positive energy during the mindfulness process.<br>**WHO-CST modification**: Considering the shame-socialised culture in China, we adapted strategies from the WHO-CST by providing specific examples that parents encounter while raising an autistic child, along with coping strategies to effectively navigate these challenges. This approach assists parents in addressing the affiliate stigma associated with parenting an autistic child. By sharing relatable experiences, we aim to empower parents to manage societal perceptions and foster a supportive environment for their children.<br>**Integration of ACT and parenting skill training**: The script for mindfulness practice "Compassion for my autistic child" was designed to help parents cultivate compassion for their autistic children. It encourages parents to reflect on their child's experiences and consider how they might feel in similar situations. Furthermore, parents practised active listening when their child expressed thoughts or feelings, which involved giving full attention, validating their emotions, and responding thoughtfully. This enables them to respond with empathy, using phrases such as, "I can see you're feeling upset, and that's okay," which helps children feel acknowledged. In addition, the practice fosters an environment where children's mistakes or emotional and behavioural problems are seen as opportunities for learning rather than failures. The integration of ACT with parenting skills training, equips parents to cultivate empathy, actively listen, and

Theme [Time & Duration]; Delivery formats	ACT/parenting skills training core principle(s)	Aim	Key activities	The adaptation and integration of ACT/WHO-CST in the context of parenting an autistic child
				foster an environment where their children's emotional and behavioural problems are viewed as valuable opportunities for growth. Moreover, the homework associated with gratitude practices encourages parents to keep a gratitude journal, where they can jot down positive moments they experience with their child each day. This helps them appreciate the present and reinforces positive parent-child interactions.
Session 8 (booster): Review and Evaluation [Week 8 & 120 minus]; Online	All core aspects of ACT and parenting skills training	Help participants practice mindfulness and quickly focus on the class	A mindfulness exercise	-
		To help participants wrap up the key techniques and strategies of emotional management and parenting of programme	7-session recap	
		Evaluate value goals and action completion	Goals completion assessment	

Theme [Time & Duration]; Delivery formats	ACT/parenting skills training core principle(s)	Aim	Key activities	The adaptation and integration of ACT/WHO-CST in the context of parenting an autistic child
		To help participants make a plan for medium-term value-based commitment actions	Future plan and actions	
		Homework review	Review of home practice of valued action and noticing barriers	
		To help participants wrap up the key techniques and strategies of emotional management and parenting of programme	Experience sharing	
		Thank the participants for their support of the programme	Awards of completion	
