# Supplementary material for: Acceptance and Commitment Training for Parents of Children With Autism Spectrum Disorder: A Randomized Clinical Trial
Source: JAMA Netw Open. 2026 Jan 8;9(1):e2552693. doi: 10.1001/jamanetworkopen.2025.52693 (PMC12784228; doi:10.1001/jamanetworkopen.2025.52693)
Supplement: Supplement 2. — eTable 1. Study Outcome Scores of Participants and Their Children With ASD at Baseline eTable 2. Pairwise Comparisons of Mean Differences of the Outcome Scores for Both Parents and Their Children With ASD Between Groups at T1 and T2 eFigure 1. Profile Plot of Parental Stress of the Study Groups Across the 3 Time Points eFigure 2. Profile Plots of the 3 Subscales of Parental Stress of the Study Groups Across the 3 Time Points eFigure 3. Profile Plot of Parental Depressive Symptoms of the Study Groups Across the 3 Time Points eFigure 4. Profile Plot of Parental Anxiety of the Study Groups Across the 3 Time Points eFigure 5. Profile Plot of Parental Psychological Flexibility of the Study Groups Across 3 Measurements eFigure 6. Profile Plot of Parenting Competence of the Study Groups Across the 3 Time Points eFigure 7. Profile Plots of the 2 Subscales of Parenting Competence of Study Groups Across 3 Measurements eFigure 8. Profile Plot of Total Difficulties of Autistic Children’s Emotional and Behavioral Problems of the Study Groups Across the 3 Time Points eFigure 9. Profile Plots of the 8 Subscales of Autistic Children’s Emotional and Behavioural Problems of the Study Groups Across the 3 Time Points eFigure 10. The Q-Q Plot for the Total Score of Parenting Stress eFigure 11. The Q-Q Plot for the Total Score of Parental Depressive Symptoms eFigure 12. The Q-Q Plot for the Total Score of Parental Anxiety eFigure 13. The Q-Q Plot for the Total Score of Parental Psychological Flexibility eFigure 14. The Q-Q Plot for the Total Score of Parenting Competence eFigure 15. The Q-Q Plot for the Total Difficulties Score of Children’s Emotional and Behavioral Problems [file jamanetwopen-e2552693-s002.pdf]

Supplementary Online Content

Li SN, Chien WT. Acceptance and commitment training for parents of children with autism spectrum disorder: a randomized clinical trial. *JAMA Netw Open.* 2025;9(1):e2552693. doi:10.1001/jamanetworkopen.2025.52693

eTable 1. Study Outcome Scores of Participants and Their Children With ASD at Baseline
eTable 2. Pairwise Comparisons of Mean Differences of the Outcome Scores for Both Parents and Their Children With ASD Between Groups at T1 and T2
eFigure 1. Profile Plot of Parental Stress of the Study Groups Across the 3 Time Points
eFigure 2. Profile Plots of the 3 Subscales of Parental Stress of the Study Groups Across the 3 Time Points
eFigure 3. Profile Plot of Parental Depressive Symptoms of the Study Groups Across the 3 Time Points
eFigure 4. Profile Plot of Parental Anxiety of the Study Groups Across the 3 Time Points
eFigure 5. Profile Plot of Parental Psychological Flexibility of the Study Groups Across 3 Measurements
eFigure 6. Profile Plot of Parenting Competence of the Study Groups Across the 3 Time Points
eFigure 7. Profile Plots of the 2 Subscales of Parenting Competence of Study Groups Across 3 Measurements
eFigure 8. Profile Plot of Total Difficulties of Autistic Children's Emotional and Behavioral Problems of the Study Groups Across the 3 Time Points
eFigure 9. Profile Plots of the 8 Subscales of Autistic Children's Emotional and Behavioural Problems of the Study Groups Across the 3 Time Points
eFigure 10. The Q-Q Plot for the Total Score of Parenting Stress
eFigure 11. The Q-Q Plot for the Total Score of Parental Depressive Symptoms
eFigure 12. The Q-Q Plot for the Total Score of Parental Anxiety
eFigure 13. The Q-Q Plot for the Total Score of Parental Psychological Flexibility
eFigure 14. The Q-Q Plot for the Total Score of Parenting Competence
eFigure 15. The Q-Q Plot for the Total Difficulties Score of Children's Emotional and Behavioral Problems

This supplementary material has been provided by the authors to give readers additional information about their work.

© 2026 Li SN and Chien WT. *JAMA Network Open*.

eTable 1. Study Outcome Scores of Participants and Their Children With ASD at Baseline

	Total (n=154) Mean (SD)	ACT-based parenting programme (n=77) Mean (SD)	Usual care (n=77) Mean (SD)	Independent t-test value	p
**PSI_SF: Total score**	43.11 (10.76)	41.79 (10.62)	44.43 (10.81)	1.53	0.13
PSI_SF: Distress	13.69 (4.25)	13.22 (4.16)	14.16 (4.31)	1.37	0.17
PSI_SF: Parent–child dysfunctional interaction	12.38 (4.13)	11.92 (3.95)	12.84 (4.27)	1.39	0.17
PSI_SF: Difficult child	17.04 (4.77)	16.65 (4.66)	17.43 (4.88)	1.01	0.31
**PHQ-9: Total score**	8.03 (5.40)	7.58 (4.83)	8.48 (5.92)	1.03	0.31
**GAD-7: Total score**	6.44 (4.74)	6.05 (4.37)	6.83 (5.08)	1.02	0.31
**Psy-Flex: Total score**	20.32 (4.66)	20.92 9(4.59)	19.73 (4.69)	-1.60	0.11
**PSOC: Total score**	62.08 (14.33)	62.92 (16.04)	61.25 (12.45)	-0.72	0.47
PSOC: Efficacy	32.60 (7.47)	32.53 (7.80)	32.68 (7.17)	0.12	0.91
PSOC: Satisfaction	29.48 (10.26)	30.39 (11.04)	28.57 (9.39)	-1.10	0.27
**SDQ**					
SDQ: Total difficulties	22.41 (6.12)	21.87 (6.17)	22.95 (6.06)	1.09	0.28
SDQ: Emotional problems	3.81 (2.53)	3.64 (2.43)	3.97 (2.62)	0.83	0.41
SDQ: Conduct problems	6.11 (2.38)	6.08 (2.51)	6.14 (2.26)	0.17	0.87
SDQ: Hyperactivity	6.64 (2.02)	6.43 (2.10)	6.84 (1.92)	1.28	0.20
SDQ: Peer problems	5.86 (2.36)	5.73 (2.28)	5.99 (2.45)	0.68	0.50
SDQ: Prosocial	4.10 (2.73)	4.22 (2.77)	3.97 (2.71)	-0.56	0.58
SDQ: Externalising	12.75	12.51 (4.54)	12.99	0.69	0.49

© 2026 Li SN and Chien WT. *JAMA Network Open*.

	Total (n=154)	ACT-based parenting programme (n=77)	Usual care (n=77)	Independent t-test value	p
	Mean (SD)	Mean (SD)	Mean (SD)		
score	(4.32)		(4.10)		
SDQ: Internalising score	9.66 (3.87)	9.36 (3.51)	9.96 (4.21)	0.96	0.34

Note. PSI-SF, Parenting Stress Index – Short Form; PHQ-9, Patient Health Questionnaire; GAD-7, Generalised Anxiety Disorder-7; PSOC, Parenting Sense of Competence Scale; SDQ, Strengths and Difficulties Questionnaire.

© 2026 Li SN and Chien WT. *JAMA Network Open*.

eTable 2. Pairwise Comparisons of Mean Differences of the Outcome Scores for Both Parents and Their Children With ASD Between Groups at T1 and T2

	**T1 Mean (SD)**		**Crude model**		**Adjusted model**		**Cohen's d#**	**T2 Mean (SD)**		**Crude model**		**Adjusted model**		**Cohen's d**
	IG	CG	Mean diff (95%CI)	*p*	Mean diff (95%CI)	*p*		IG	CG	Mean diff (95%CI)	*p*	Mean diff (95%CI)	*p*	
**PSI_SF: Total score**	36.65 (8.77)	44.49 (9.73)	7.84 (4.92, 10.77)	***<0.001***	7.45 (4.35, 10.55)	***<0.001***	0.85	36.95 (8.26)	43.66 (10.74)	6.71 (3.70, 9.73)	***0.001***	6.32 (3.08, 9.56)	***<0.001***	0.70
PSI_SF: Distress	11.60 (3.23)	13.84 (4.36)	2.25 (1.04, 3.45)	***<0.001***	2.20 (0.93, 3.46)	***<0.001***	0.58	11.60 (3.40)	13.48 (4.30)	-	-	-	-	-
PSI_SF: Parent–child dysfunctional interaction	10.05 (3.26)	12.70 (3.44)	2.65 (1.60, 3.70)	***<0.001***	2.50 (1.42, 3.58)	***<0.001***	0.79	10.45 (3.07)	12.78 (3.52)	2.33 (1.29, 3.36)	***<0.001***	2.17 (1.09, 3.25)	***<0.001***	0.71
PSI_SF: Difficult child	15.00 (4.12)	17.95 (4.40)	2.95 (1.61, 4.28)	***<0.001***	2.76 (1.35, 4.17)	***<0.001***	0.69	14.90 (3.95)	17.40 (4.80)	2.51 (1.13, 3.89)	***<0.001***	2.32 (0.87, 3.77)	***0.002***	0.57
**PHQ-9: Total score**	5.81 (4.60)	8.31 (6.26)	2.51 (0.78, 4.23)	***0.005***	2.47 (0.74, 4.20)	***0.005***	0.46	5.81 (4.64)	8.17 (6.10)	-	-	-	-	-

© 2026 Li SN and Chien WT. *JAMA Network Open*.

	T1 Mean (SD)		Crude model		Adjusted model		Cohen's d#	T2 Mean (SD)		Crude model		Adjusted model		Cohen's d
	IG	CG	Mean diff (95%CI)	*p*	Mean diff (95%CI)	*p*		IG	CG	Mean diff (95%CI)	*p*	Mean diff (95%CI)	*p*	
**GAD-7: Total score**	4.00 (3.82)	6.40 (5.75)	2.40 (0.87, 3.94)	***0.002***	2.74 (1.18, 4.30)	***<0.001***	0.49	4.94 (5.15)	6.87 (5.09)	-	-	-	-	-
**Psy-Flex: Total score**	22.45 (3.57)	19.62 (4.29)	-2.83 (-4.07, -1.59)	***<0.001***	-2.88 (-4.18, -1.58)	***<0.001***	0.72	23.13 (3.53)	19.69 (4.06)	-3.44 (-4.64, -2.25)	***<0.001***	-3.49 (-4.76, -2.23)	***<0.001***	0.90
**PSOC: Total score**	67.90 (11.40)	61.14 (11.13)	-6.75 (-10.29, -3.22)	***<0.001***	-7.04 (-10.93, -3.15)	***<0.001***	0.60	68.21 (10.58)	61.64 (10.28)	-6.57 (-9.84, -3.30)	***<0.001***	-6.86 (-10.42, -3.29)	***<0.001***	0.63
PSOC: Efficacy	34.83 (5.21)	32.64 (5.50)	-2.20 (-3.87, -0.52)	***0.011***	-2.47 (-4.25, -0.74)	***0.006***	0.41	35.32 (4.64)	32.95 (5.29)	-2.38 (-3.94, -0.82)	***0.003***	-2.66 (-4.33, -0.98)	***0.002***	0.48
PSOC: Satisfaction	33.06 (7.82)	28.51 (7.99)	3.86 (2.35, 5.36)	***<0.001***	-4.60 (-7.39, -1.82)	***0.001***	0.58	32.88 (7.57)	28.69 (7.62)	-	-	-	-	-
**SDQ**														
SDQ: Total difficulties	19.78 (5.83)	23.12 (4.52)	3.34 (1.70, 4.98)	***<0.001***	3.56 (1.81, 5.32)	***<0.001***	0.64	19.32 (7.04)	22.71 (5.17)	3.39 (1.45, 5.33)	***<0.001***	3.61 (1.56, 5.66)	***<0.001***	0.55
SDQ:	3.05	4.23	1.18	***0.001***	1.38	***<0.001***	0.53	2.69	3.77	-	-	-	-	-

© 2026 Li SN and Chien WT. *JAMA Network Open*.

	T1 Mean (SD)		Crude model		Adjusted model		Cohen's d#	T2 Mean (SD)		Crude model		Adjusted model		Cohen's d
	IG	CG	Mean diff (95%CI)	*p*	Mean diff (95%CI)	*p*		IG	CG	Mean diff (95%CI)	*p*	Mean diff (95%CI)	*p*	
Emotional problems	(2.33)	(2.13)	(0.48, 1.88)		(0.67, 2.10)			(1.76)	(2.33)					
SDQ: Conduct problems	5.08 (2.14)	5.97 (1.62)	0.90 (0.30, 1.49)	***0.003***	0.92 (0.27, 1.57)	***0.006***	0.67	5.25 (2.59)	6.16 (1.47)	0.91 (0.25, 1.57)	0.0070	0.93 (0.22, 1.64)	***0.010***	0.43
SDQ: Hyperactivity	5.84 (1.91)	6.88 (1.93)	1.04 (0.44, 1.64)	***<0.001***	1.00 (0.37, 1.63)	***0.002***	0.54	6.10 (2.08)	6.92 (1.76)	-	-	-	-	-
SDQ: Peer problems	5.81 (1.97)	6.03 (1.81)	-	-	-	-	-	5.29 (1.99)	5.87 (1.55)	-	-	-	-	-
SDQ: Prosocial	4.56 (1.96)	4.05 (2.09)	-	-	-	-	-	4.29 (2.33)	4.10 (2.46)	-	-	-	-	-
SDQ: Externalising	10.92 (3.35)	12.86 (2.83)	1.94 (0.96, 2.91)	***<0.001***	1.92 (0.86, 2.98)	***<0.001***	0.63	11.35 (4.28)	13.08 (2.37)	-	-	-	-	-
SDQ: Internalising	8.86 (3.58)	10.26 (2.98)	-	-	-	-	-	7.97 (3.54)	9.64 (3.38)	-	-	-	-	-

Notes: #Pairwise comparisons were conducted only for outcomes that had significant interactions between group and time at T1 and/or T2. PSI-SF = Parenting Stress Index – Short Form; PHQ-9 = Patient Health Questionnaire-9; GAD-7 = Generalised Anxiety Disorder-7; PSOC = Parenting Sense of Competence Scale; SDQ = Strengths and Difficulties Questionnaire; EG = experimental group; CG = control group.

© 2026 Li SN and Chien WT. *JAMA Network Open*.

Interpretations for Supplementary eTable 2

Parental stress. Pairwise comparisons in the GEE models showed significant between-group differences in mean scores at both T1 and T2 in the crude (T1: mean difference = 7.84; 95% CI, 4.92–10.77; P < .001; T2: mean difference = 6.71; 95% CI, 3.70–9.73; P = .001) and adjusted (T1: mean difference = 7.45; 95% CI, 4.35–10.55; P < .001; T2: mean difference = 5.92; 95% CI, 3.08–9.56; P < .001) models, with a large effect at T1 (Cohen d = 0.85) and a medium effect at T2 (d = 0.70). The pairwise comparisons of the parent–child dysfunctional interaction and difficult child subscales showed significant differences in mean scores at both T1 and T2, with medium effect sizes (d ranged from 0.57 to 0.79). And the pairwise comparisons of the distress subscale showed significant differences in mean scores at T1, with medium effect size (d=0.58).

Parental depressive symptoms. Pairwise comparisons in the GEE models showed significant between-group differences in mean scores at T1 in both the crude (mean difference = 2.51; 95% CI, 0.78–4.23; P = .005) and adjusted (mean difference = 2.47; 95% CI, 0.74–4.20; P = .005) models, with a small effect (d = 0.46). The hypothesis that the intervention group would have a greater decrease in depressive symptoms than the control group at T2 was not supported.

Parental anxiety. Pairwise comparisons in the GEE models (Tables 8–12) showed significant between-group differences in mean scores at T1, with results from the crude model (T1: mean difference = 2.40; 95% CI, 0.87–3.94; P = .002; T2: mean difference = 1.94; 95% CI, 0.33–3.54; P = .019) and the adjusted model (T1: mean difference = 2.74; 95% CI, 1.18–4.30; P < .001; T2: mean difference = 2.27; 95% CI, 0.62–3.93; P = .008), with a small effect (d = 0.49).

Parental psychological flexibility. Pairwise comparisons in the GEE models showed significant between-group differences in mean scores at both T1 and T2 in the crude (T1: mean difference = −2.83; 95% CI, −4.07 to −1.59; P < .001; T2: mean difference = −3.44; 95% CI, −4.64 to −2.25; P < .001) and adjusted (T1: mean difference = −2.88; 95% CI, −4.18 to −1.58; P < .001; T2: mean difference = −3.49; 95% CI, −4.76 to −2.23; P < .001) models, with medium-to-large effects at T1 (Cohen d = 0.72) and T2 (d = 0.90).

Parental parenting competence. Pairwise comparisons in the GEE models showed significant between-group differences in mean scores at both T1 and T2 in the crude (T1: mean difference = −6.75; 95% CI, −10.29 to −3.22; P < .001; T2: mean difference = −6.57; 95% CI, −9.84 to −3.30; P < .001) and adjusted (T1: mean difference = −7.04; 95% CI, −10.93 to −3.15; P < .001; T2: mean difference = −6.86; 95% CI, −10.42 to −3.29; P < .001) models, with medium effects at T1 (Cohen d = 0.60) and T2 (d = 0.63). For the efficacy subscale, pairwise comparisons showed significant differences at both T1 (crude: mean difference = −2.20; 95% CI, −3.87 to −0.52; P = .011; adjusted: mean difference = −2.47; 95% CI, −4.25 to −0.74; P = .006) and T2 (crude: mean difference = −2.38; 95% CI, −3.98 to −0.82; P = .003; adjusted: mean difference = −2.66; 95% CI, −4.33 to

© 2026 Li SN and Chien WT. *JAMA Network Open*.

−0.98; P = .002), with small effects at T1 (d = 0.41) and T2 (d = 0.48). For the satisfaction subscale, pairwise comparisons showed a significant difference at T1 (crude: mean difference = 3.86; 95% CI, 2.35–5.36; P < .001; adjusted: mean difference = −4.60; 95% CI, −7.39 to −1.82; P = .001), with a medium effect (d = 0.58).

Children's emotional and behavioral problems. Pairwise comparisons in the GEE models showed significant between-group differences in mean scores at both T1 and T2 in the crude (T1: mean difference = 3.34; 95% CI, 1.70–4.98; P < .001; T2: mean difference = 3.39; 95% CI, 1.45–5.33; P < .001) and adjusted (T1: mean difference = 3.56; 95% CI, 1.81–5.32; P < .001; T2: mean difference = 3.61; 95% CI, 1.56–5.66; P < .001) models, with medium effects at T1 (d = 0.64) and T2 (d = 0.55). For total difficulties and conduct problems, pairwise comparisons showed significant differences at T1 and T2 in both models (mean differences, 0.90–3.34; all P < .05), with small-to-medium effects (d, 0.43–0.67). For emotional problems, hyperactivity, and externalizing, pairwise comparisons showed significant differences at T1 in both models (mean differences, 1.04–1.94; all P < .05), with medium effects (d, 0.53–0.64). There were no significant differences for peer problems, prosocial behavior, or internalizing at either T1 or T2 in either the crude or adjusted models.

© 2026 Li SN and Chien WT. *JAMA Network Open*.

eFigure 1. Profile Plot of Parental Stress of the Study Groups Across the 3 Time Points

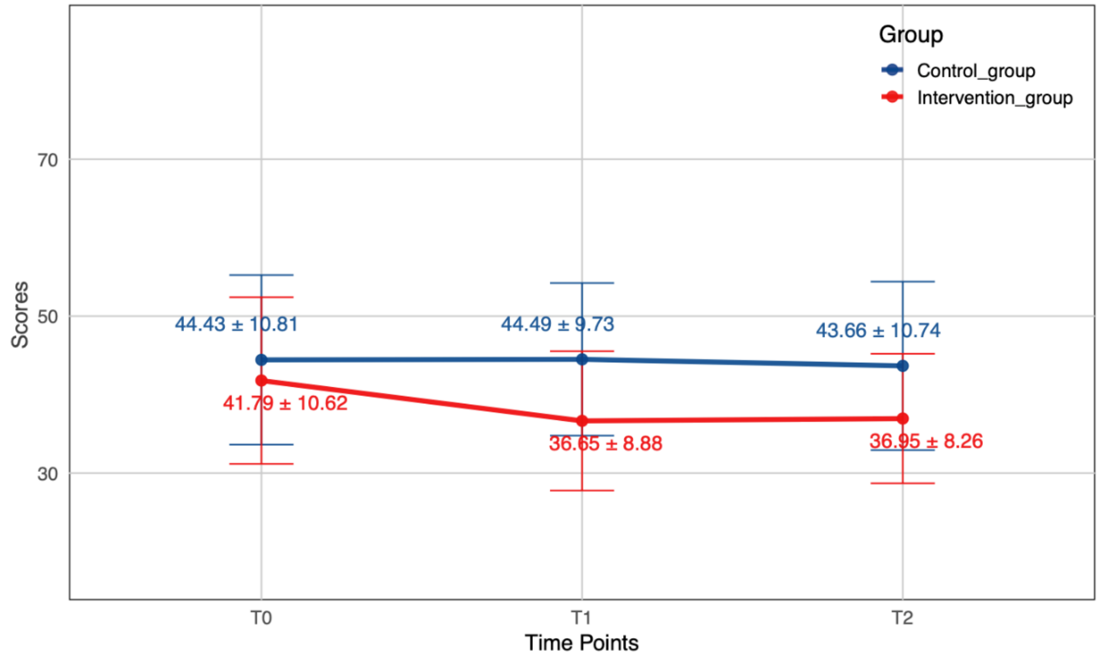

Note. Scores at each time point represent the mean score ± standard deviation, and the error bars represent the standard deviation for each group at those time points.

eFigure 2. Profile Plots of the 3 Subscales of Parental Stress of the Study Groups Across the 3 Time Points

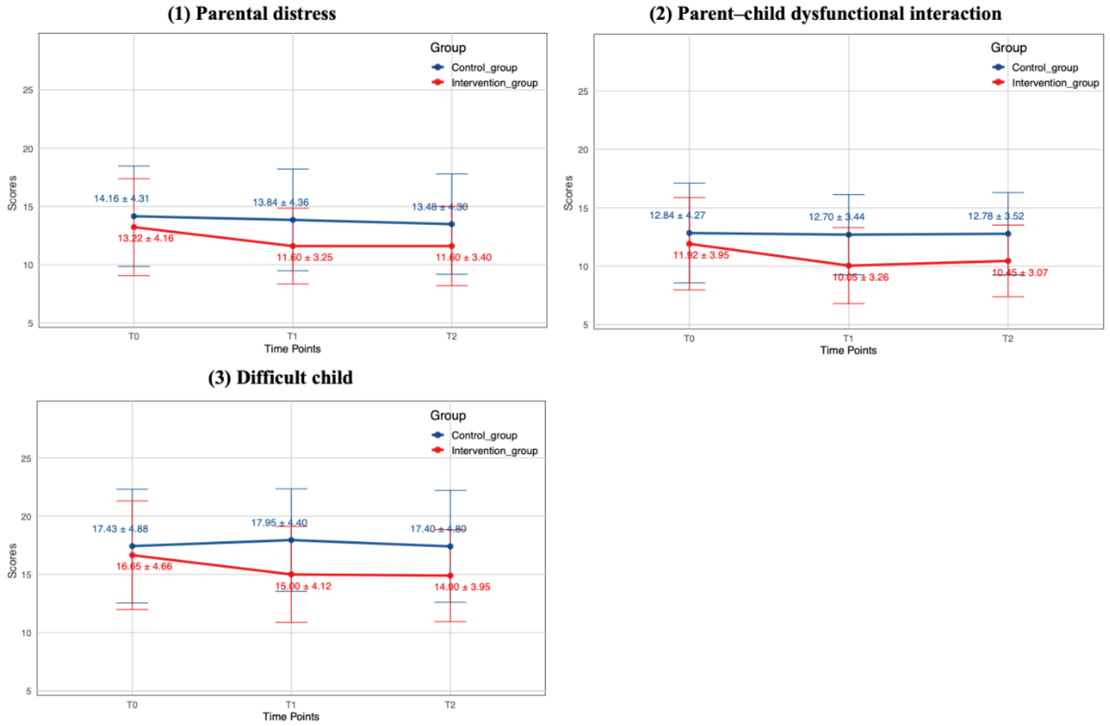

© 2026 Li SN and Chien WT. *JAMA Network Open*.

Note. Scores at each time point represent the mean score ± standard deviation, and the error bars represent the standard deviation for each group at those time points.

eFigure 3. Profile Plot of Parental Depressive Symptoms of the Study Groups Across the 3 Time Points

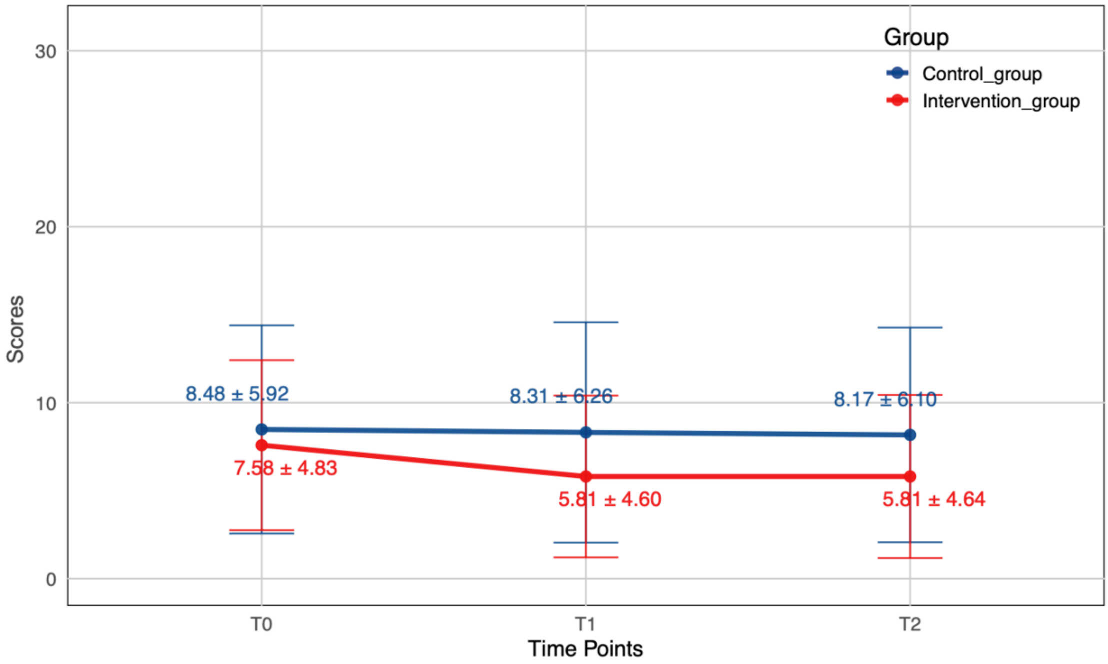

Note. Scores at each time point represent the mean score ± standard deviation, and the error bars represent the standard deviation for each group at those time points.

© 2026 Li SN and Chien WT. *JAMA Network Open*.

eFigure 4. Profile Plot of Parental Anxiety of the Study Groups Across the 3 Time Points

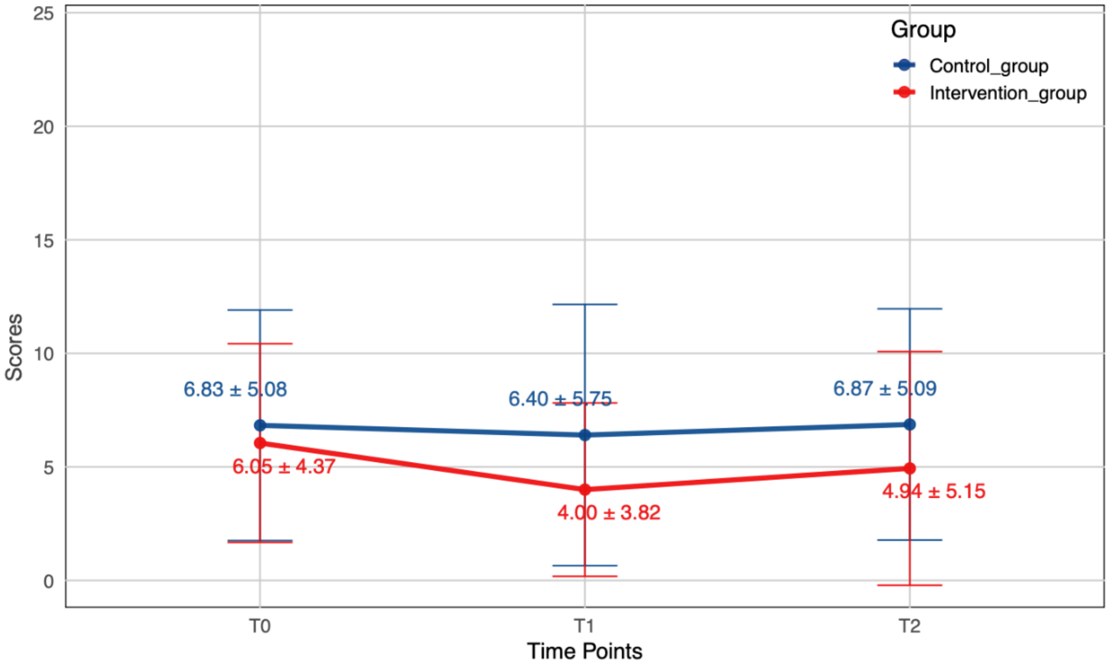

Note. Scores at each time point represent the mean score, and the error bars represent the standard deviation for each group at those time points.

© 2026 Li SN and Chien WT. *JAMA Network Open*.

eFigure 5. Profile Plot of Parental Psychological Flexibility of the Study Groups Across 3 Measurements

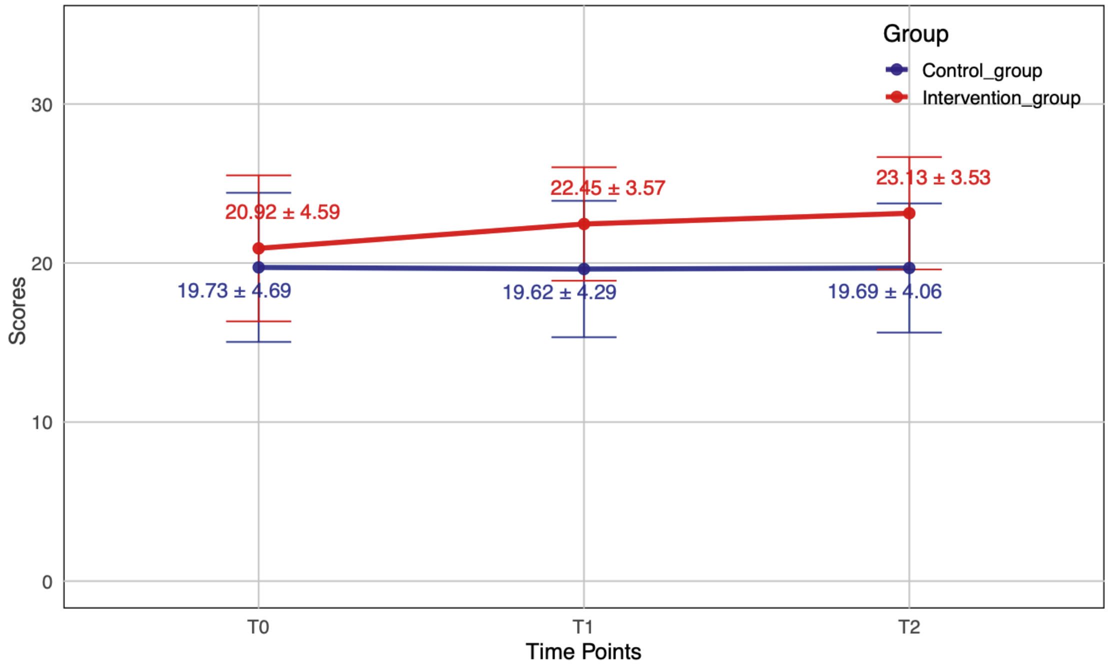

Note. Scores at each time point represented the mean score ± standard deviation; and the error bars represent the standard deviations for each group.

eFigure 6. Profile Plot of Parenting Competence of the Study Groups Across the 3 Time Points

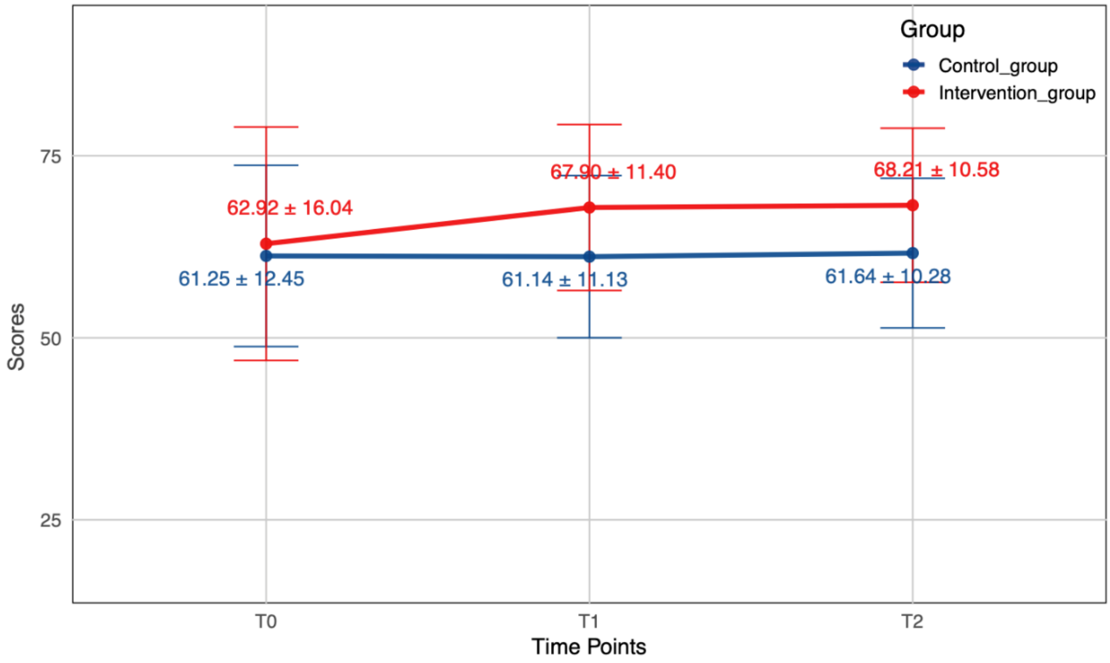

Note. Scores at each time point represented the mean score ± standard deviation of the group; and the error bars represented the standard deviation of each group.

© 2026 Li SN and Chien WT. *JAMA Network Open*.

eFigure 7. Profile Plots of the 2 Subscales of Parenting Competence of Study Groups Across 3 Measurements

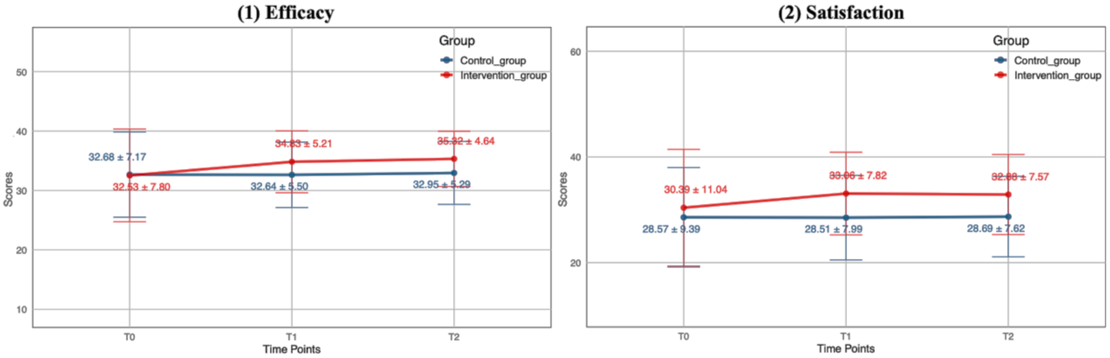

Note. Scores at each time point represented the mean score ± standard deviation; and the error bars represented the standard deviations of each group.

eFigure 8. Profile Plot of Total Difficulties of Autistic Children’s Emotional and Behavioral Problems of the Study Groups Across the 3 Time Points

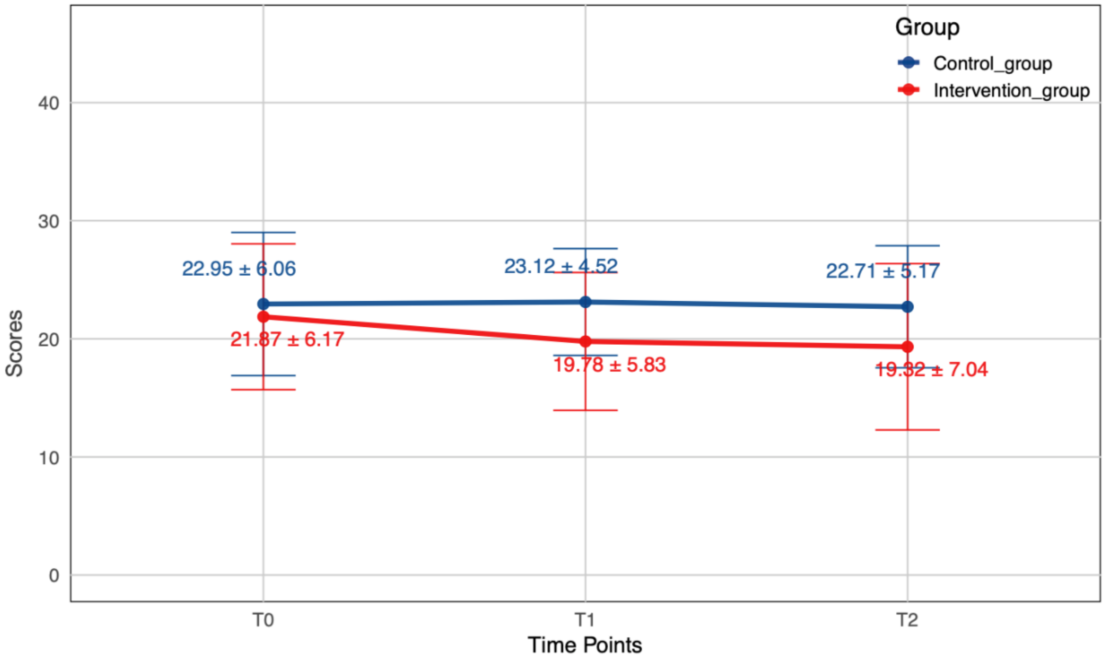

Note. Scores at each time point represent the mean score ± standard deviation, and the error bars represent the standard deviation for each group at those time points.

© 2026 Li SN and Chien WT. *JAMA Network Open*.

eFigure 9. Profile Plots of the 8 Subscales of Autistic Children's Emotional and Behavioural Problems of the Study Groups Across the 3 Time Points

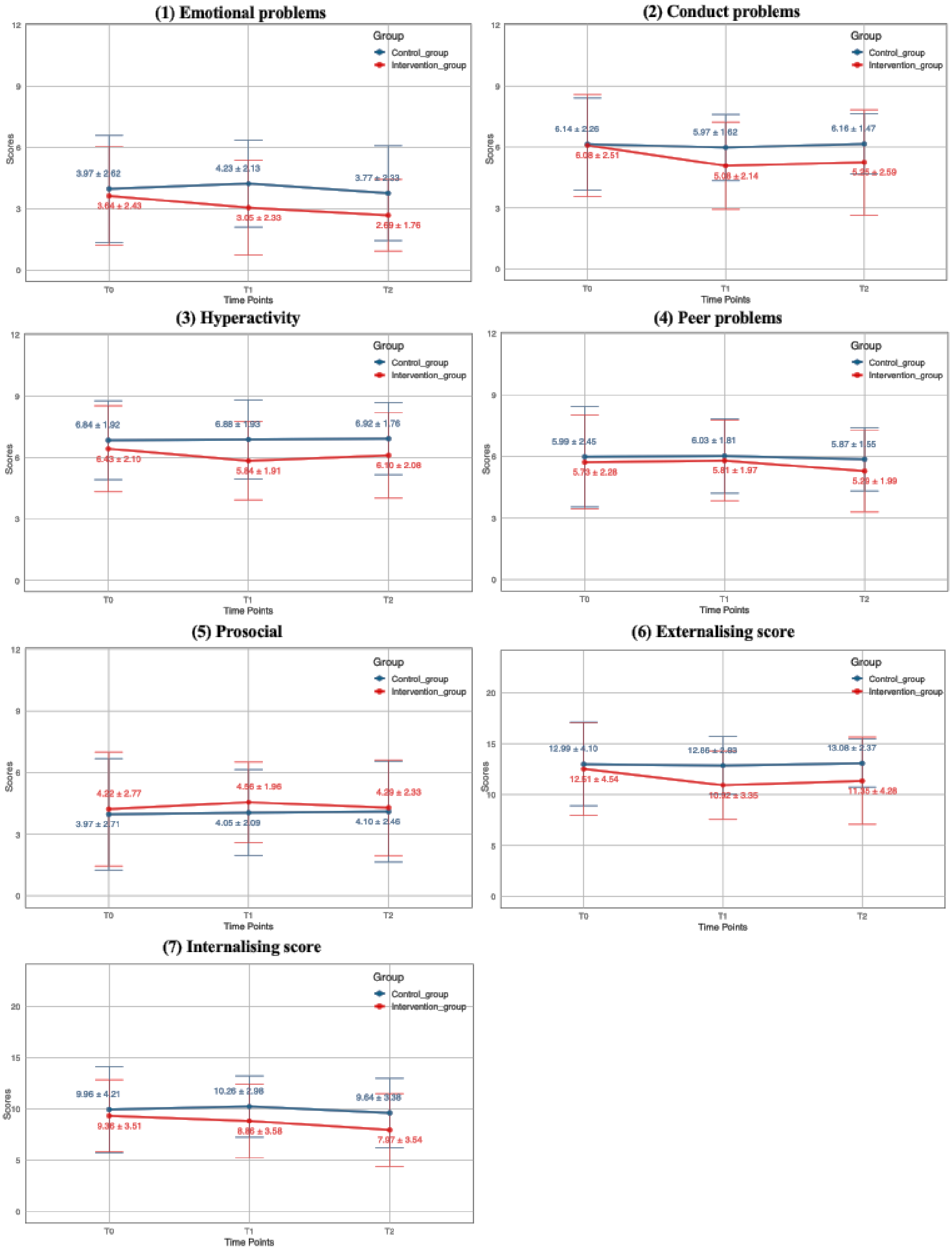

Note. Scores at each time point represent the mean score ± standard deviation, and the error bars represent the standard deviation for each group at those time points

© 2026 Li SN and Chien WT. *JAMA Network Open*.

eFigure 10. The Q-Q Plot for the Total Score of Parenting Stress

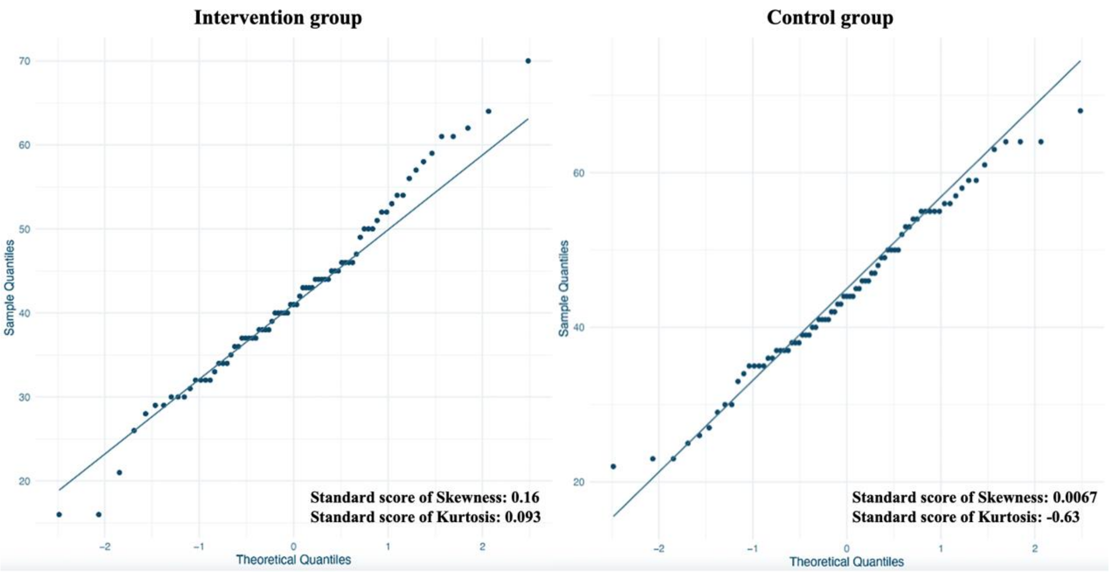

eFigure 11. The Q-Q Plot for the Total Score of Parental Depressive Symptoms

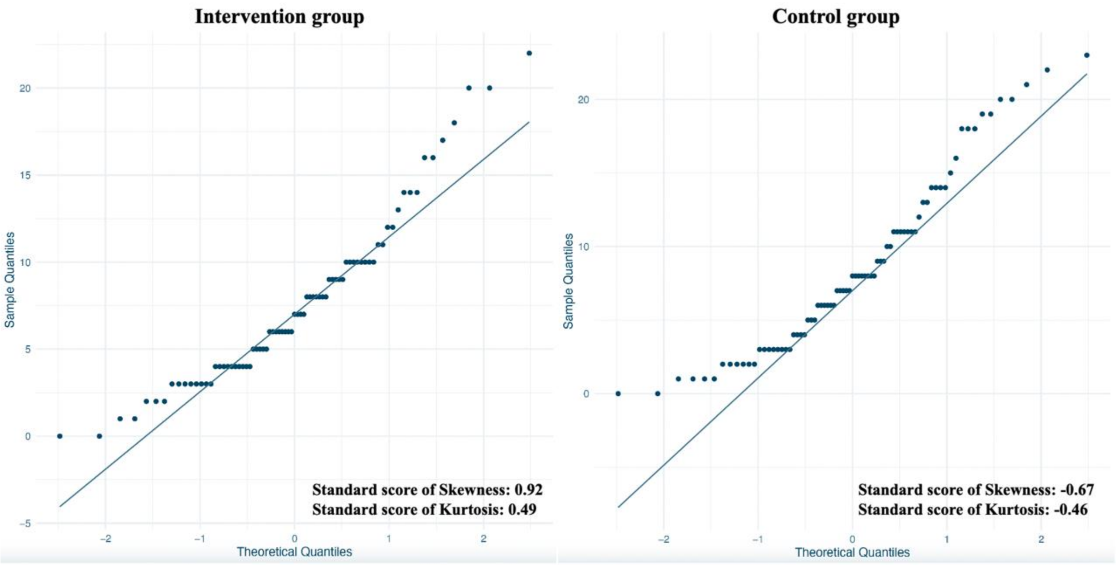

© 2026 Li SN and Chien WT. *JAMA Network Open*.

eFigure 12. The Q-Q Plot for the Total Score of Parental Anxiety

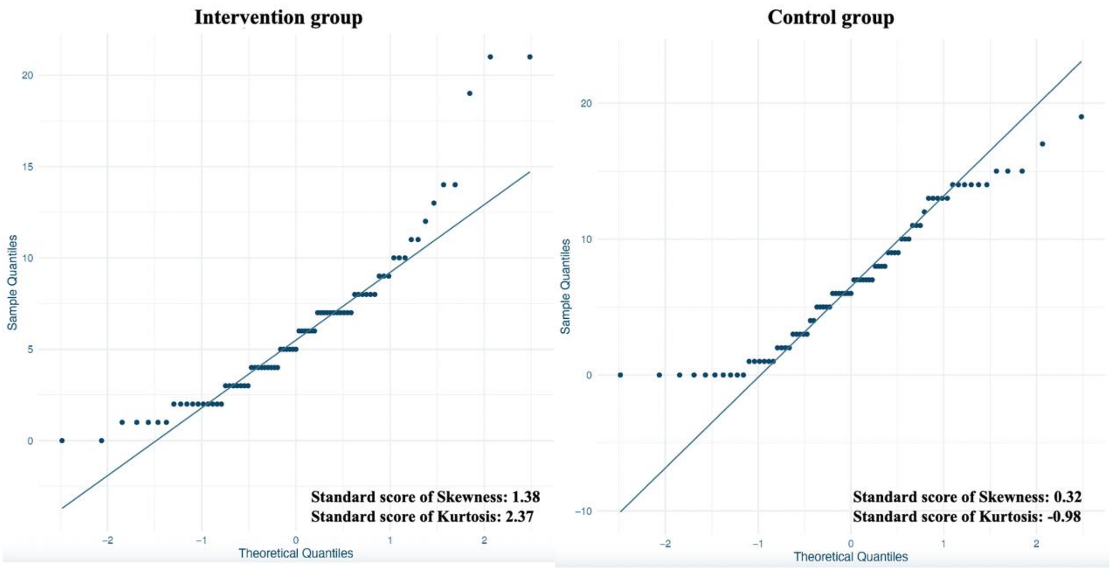

eFigure 13. The Q-Q Plot for the Total Score of Parental Psychological Flexibility

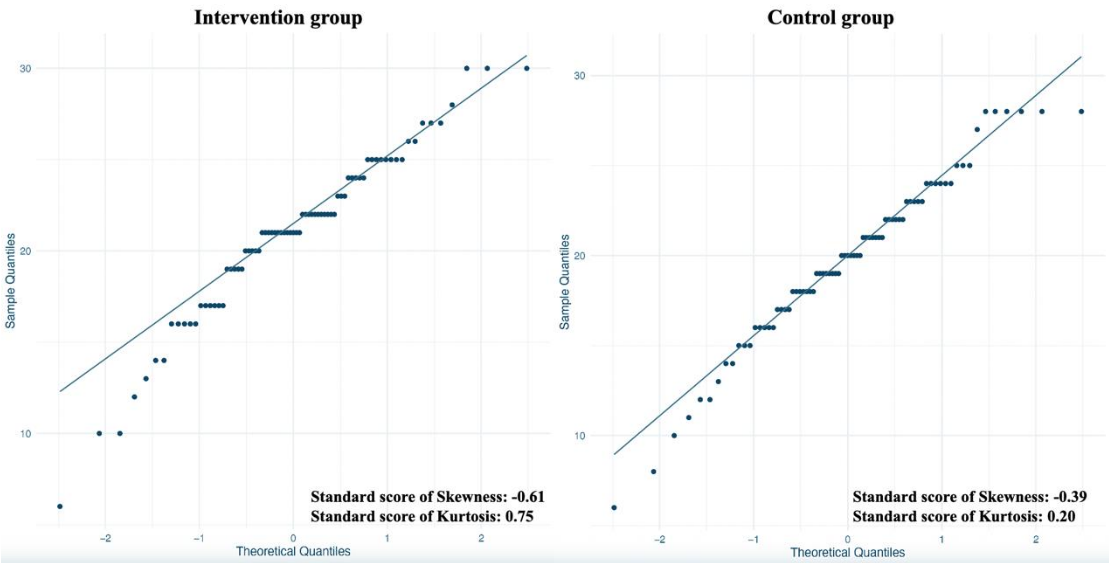

© 2026 Li SN and Chien WT. *JAMA Network Open*.

eFigure 14. The Q-Q Plot for the Total Score of Parenting Competence

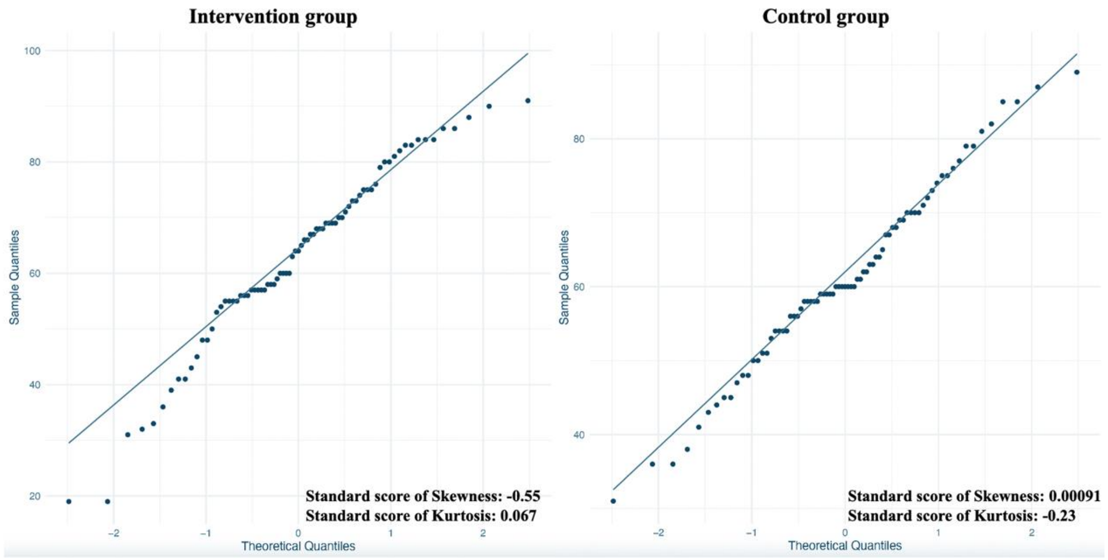

eFigure 15. The Q-Q Plot for the Total Difficulties Score of Children's Emotional and Behavioral Problems

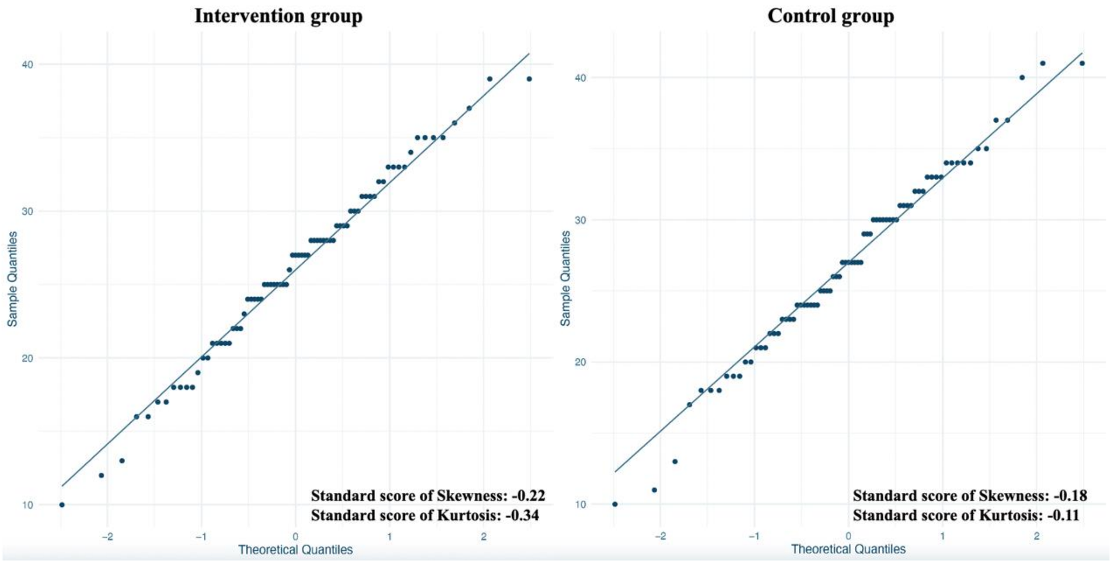

© 2026 Li SN and Chien WT. *JAMA Network Open*.
