# Supplementary material for: Acceptance and Commitment Training for Parents of Children With Autism Spectrum Disorder: A Randomized Clinical Trial
Source: JAMA Netw Open. 2026 Jan 8;9(1):e2552693. doi: 10.1001/jamanetworkopen.2025.52693 (PMC12784228; doi:10.1001/jamanetworkopen.2025.52693)
Supplement: Supplement 3. — Data Sharing Statement [file jamanetwopen-e2552693-s003.pdf]

Data Sharing Statement

Li. Acceptance and Commitment Training for Parents of Children With Autism Spectrum Disorder. *JAMA Netw Open*. Published January 08, 2026.
doi:10.1001/jamanetworkopen.2025.52693

Data
Additional Information: Chinese Clinical Trial Registry identifier: ChiCTR2400080472 (URL:https://www.chictr.org.cn/indexEN.html)
Data available: No

Additional Information
Explanation for why data not available: The datasets generated and/or analysed during the current study are not publicly available due to the need to maintain anonymity of participants and the confidentiality of the data. However, the datasets were available from the corresponding authors on reasonable request.
